# Associations of Triglycerides and Atherogenic Index of Plasma with Brain Structure in the Middle-Aged and Elderly Adults

**DOI:** 10.3390/nu16050672

**Published:** 2024-02-27

**Authors:** Xixi Chen, Yujia Bao, Jiahao Zhao, Ziyue Wang, Qijing Gao, Mingyang Ma, Ziwen Xie, Mu He, Xiaobei Deng, Jinjun Ran

**Affiliations:** 1 School of Public Health, Shanghai Jiao Tong University School of Medicine, Shanghai 200025, China; c.cctx@sjtu.edu.cn (X.C.); bubble-y@sjtu.edu.cn (Y.B.); wangziyue@sjtu.edu.cn (Z.W.); gqjjj0729@sjtu.edu.cn (Q.G.); mmy1313@sjtu.edu.cn (M.M.); shuji888@sjtu.edu.cn (Z.X.); dengxiaobei@sjtu.edu.cn (X.D.); 2 Department of Foundational Mathematics, Xi’an Jiaotong-Liverpool University, Suzhou 215000, China; jiahao.zhao22@student.xjtlu.edu.cn

**Keywords:** triglyceride, atherogenic index of plasma, brain imaging trait, molecular epidemiology

## Abstract

Triglyceride (TG) and atherogenic index of plasma (AIP) have been acknowledged to be risk factors for vascular insults, but their impacts on the brain system remain elusive. To fill in some gaps, we investigated associations of TG and AIP with brain structure, leveraging the UK Biobank database. TG and high-density lipoprotein cholesterol (HDL-C) were examined at baseline and AIP was calculated as log (TG/HDL-C). We build several linear regression models to estimate associations of TG and AIP with volumes of brain grey matter phenotypes. Significant inverse associations of TG and AIP with volumes of specific subcortical traits were observed, among which TG and AIP were most significantly associated with caudate nucleus (TG: β [95% confidence interval CI] = −0.036 [−0.051, −0.022], AIP: −0.038 [−0.053, −0.023]), thalamus (−0.029 [−0.042, −0.017], −0.032 [−0.045, −0.019]). Higher TG and AIP were also considerably related with reduced cortical structure volumes, where two most significant associations of TG and AIP were with insula (TG: −0.035 [−0.048, −0.022], AIP: −0.038 [−0.052, −0.025]), superior temporal gyrus (−0.030 [−0.043, −0.017], −0.033 [−0.047, −0.020]). Modification effects of sex and regular physical activity on the associations were discovered as well. Our findings show adverse associations of TG and AIP with grey matter volumes, which has essential public health implications for early prevention in neurodegenerative diseases.

## 1. Introduction

Given the prominent socio-economic burden caused by the about 15% to 55% increase in disability-adjusted life-years, neuro-psychiatric diseases have become a significant focus globally [1,2]. Vascular lesions that confer early risk for brain disorders are known to play an essential role in their etiology [3,4]. Lipids are abundant in the heart-brain plasma; these were deemed to be critical indicators of atherogenicity and play a significant role in brain disorders [4], implying that abnormal lipid levels may have a potential impact on brain structure. Brain magnetic resonance imaging (MRI) was recognized as an underlying method for predicting neuro-psychiatric diseases due to its role in indicating and quantifying brain structure. Thus, by assessing its relationship with alterations of brain structure, lipid traits are potentially promoted as the preliminary biomarker to predict early risks of brain disorders.

Recent studies, with limited sample sizes, on dyslipidemia and brain structure alterations demonstrated inconsistent results. It was documented that circulatory cholesterol was associated with grey matter atrophy in partial observations [5,6], and reduced cortical thickness was discovered in individuals with metabolic syndrome [7]. Nevertheless, other studies observed associations of higher levels of total cholesterol (TC), high-density lipoprotein cholesterol (HDL-C), and triglyceride (TG) with abnormal signals in grey matter volumes [8]. The relationship of elevated cholesterol and higher cortical thickness has also been reported [9,10]. A high level of TG accompanied by decreased HDL-C was shown to be a critical factor in vascular pathology [11]. Atherogenic index of plasma (AIP), as a link between protective and atherogenic lipoproteins, could be a better indicator for evaluating brain structure defects and identifying early risks of brain disorders than a single lipid trait [12]. To date, evidence about the impacts of TG and AIP on specific brain imaging traits, is still limited, which highly impeded the public understanding of molecular epidemiological mechanisms and the development of preventive interventions on the pathway from lipids to brain disorders.

Therefore, we evaluated epidemiological associations of TG and AIP with brain imaging phenotypes based on a large longitudinal cohort in the UK Biobank to figure out which specific regions were affected by dyslipidemia. We further estimated the effect modifications of age, sex, waist-to-hip ratio (WHR), healthy sleep pattern, and regular physical activity on the associations. Our study offers new perspectives on preventing brain-related diseases through lipid interventions.

## 2. Materials and Methods

### 2.1. Study Population

UK Biobank is a longitudinal prospective cohort in which more than half a million individuals aged 40 to 69 years were enrolled in the UK between 2006 and 2010. The UK Biobank contributes to enhancing understanding of the prevention and diagnosis of and cures for serious illnesses. Baseline data were collected at 22 assessment centers in England, Scotland, and Wales, encompassing information on population characteristics, lifestyle, health conditions, and biological samples. In addition to physical examinations, surveys, and concise interviews, enhancement data were collected via online questionnaires. In 2014, UK Biobank collected imaging study data, including abdominal, brain, and heart MRI scans, carotid ultrasound scans, and 12-lead electrocardiogram measurements, all taken at the Imaging Assessment Centre. The UK Biobank obtained ethical approval from the North West Multi-Centre Research Ethics Committee (Reference: 11/NW/03820). Our study was conducted under Application Number 99001 (Approval Code), and the approval date ranges from 31 January 2023 to 31 January 2026. Participants who withdrew from the cohort study were not eligible for the analyses. A total of 25,057 individuals remained in the analyses after excluding 177,636 participants who were missing complete data on lipid biomarkers (TG and HDL-C) and covariates, 296,384 without brain imaging data, and 3292 prevalent cases with lipid-lowering drugs at baseline (statin, ezetimibe, fibrate, niacin, and others). This study conformed to the Strengthening the Reporting of Observational Studies in Epidemiology guideline.

### 2.2. Lipid-Related Exposures

The serum lipid indicators selected in our study were TG and AIP. AIP was obtained by logarithmically converting TG to HDL-C ratio with the base of 10: log (TG/HDL-C) [12]. We extracted TG (field code 30870) and HDL-C (field code 30760) from UK Biobank Blood assays (category 10080) and Blood biochemistry (category 18518) Biological samples datasets. TG and HDL-C were measured by GPO-POD analysis and enzyme immunoinhibition analysis using a Beckman Coulter AU5800 in the UK Biobank. TG was log-transformed before modeling. Both categorical (divided into quarters) and continuous (per SD) TG were analyzed, and so were AIP.

### 2.3. Outcomes

For the current analyses, we utilized varieties of brain imaging phenotypes in the UK Biobank as outcomes, which were acquired from quality-controlling T1-weighted imaging. The brain structure images were scanned by a standard Siemens Skyra 3T running VD13A SP4, with a standard Siemens 32-channel radio frequency receive head coil. During MRI pre-processing, brain structure was segmented into grey matter, white matter, and cerebrospinal fluid (CSF), each of which were anatomically registered and ultimately normalized. For the imaging data, image-derived phenotypes were generated via FMRIB Software Library version 5.0.10 and FreeSurfer version 6.0, which can represent and quantify the structure and functions of the brain. All selected imaging data were presented in cubic millimeters (mm^3^), encompassing the volumes of 7 subcortical and 33 cortical phenotypes. These data were extracted from FreeSurfer desikan white (category 192) and subcortical volumes (FIRST) (category 1102), respectively. The data fields corresponding to each brain grey matter phenotype can be found in the online showcase of the UK Biobank accessed on 20 July 2023 (https://biobank.ctsu.ox.ac.uk/).

### 2.4. Covariates

We selected several potential confounding variables as covariates, including age, sex, index of multiple deprivation (IMD), WHR, healthy lifestyle, systolic blood pressure (SBP), diastolic blood pressure (DBP), low-density lipoprotein cholesterol (LDL-C), TC, hemoglobin A1c (HbA1c), and prevalence of hypertension, diabetes, atrial fibrillation, and stroke. IMD was a measure of relative deprivation based on the national census output area and was determined from seven domains of deprivation, including income score, employment score, health score, education score, housing score, crime score, and living environment score [13]. Compared with the Townsend Deprivation Index measured by unemployment, car ownership, household overcrowding, and owner occupation [14], IMD could provide a more comprehensive and better evaluation of socioeconomic status. WHR was calculated as waist/hip measurement and categorized into poor (≥0.9 for men and ≥0.85 for women) and ideal. 

Healthy lifestyle (never smoking, never drinking, healthy sleep, regular physical activity, and healthy diet) was also considered in the study [15]. Smoking status and alcohol intake were collected through a touchscreen questionnaire and classified as never and current or previous smoking/drinking. Healthy sleep pattern was measured by five dimensions of sleep behaviors (early chronotype, sleep 7–8 h/day, never/rarely or sometimes insomnia, no self-reported snoring, and never/rarely or sometimes excessive daytime sleepiness) [16], and was also categorized into yes (≥4 healthy components) and no. Regular physical activity was defined as at least 150 min of moderate-intensity per week or 75 min of vigorous-intensity per week, or a combination of both [17]. For healthy diet, seven healthy diet components were recommended, incorporating more than three servings of fruit daily, three servings of vegetables daily, two servings of fish weekly, three servings of whole grains daily, and less than one serving of processed meat weekly, one and a half servings of unprocessed red meat weekly, and one and a half servings of refined grains daily [18]. We categorized this into yes (≥4 healthy diet components) and no. SBP and DBP were measured using the Omron HEM-7015IT digital blood pressure monitor and the average of the two recordings was utilized in the analysis.

### 2.5. Statistical Analysis

Baseline characteristics and brain imaging phenotypes were expressed as mean and standard deviation (SD) for continuous variables and as numbers and percentages for categorical variables. All brain structural phenotypes were normalized for head size using z-transformation. Several linear regression models were built to evaluate the associations of TG and AIP with selected brain structure after adjusting for age, sex, IMD, WHR, healthy lifestyle, SBP, DBP, HbA1c, LDL-C, TC, prevalence of hypertension, diabetes, atrial fibrillation, and stroke. The results were demonstrated with βs and the corresponding 95% confidence intervals (CIs). False discovery rate (FDR) corrections were performed for the problem of multiple comparisons (α = 0.05) to reduce type Ⅰ error and were applied at the appropriate time. Participants with loss to follow-up and those missing data in exposures and outcomes were removed before analysis.

We also performed stratified analyses to test whether the associations between lipid indicators (TG and AIP) and each brain grey matter phenotype varied by age (≤64 and ≥65), sex (male and female), WHR (poor and ideal), healthy sleep (yes and no), regular activity (yes and no), and polygenic risk scores (PRS) for TG (low and high). PRS were deemed to predict genetic predisposition to diseases [19] and were categorized into low and high generic risks of TG according to the median of PRS. The statistical significance for the interaction effect was detected through the multiple linear regression model with a multiplicative term. We also conducted sensitivity analyses to appraise the reliability and robustness of conclusions and assess the effect of noncritical factors on them: (i) analyses were restricted to white British Caucasians; (ii) individuals with extremely small exposures (<1% of TG and AIP values) were excluded; (iii) participants with extremely large exposures (>99%) were excluded; (iv) analyses were performed with additional control for C-reactive protein; (v) patients with pancreatitis were excluded; (vi) analyses were performed after controlling for hypertension, heart attack, and diabetes medications; (vii) analyses were conducted after controlling for antiplatelet or anticoagulant medications. We considered *p* < 0.05 statistically significant, and statistical analyses were two-sided. The analyses were conducted with R software, version 4.2.2.

## 3. Results

**Figure 1:**
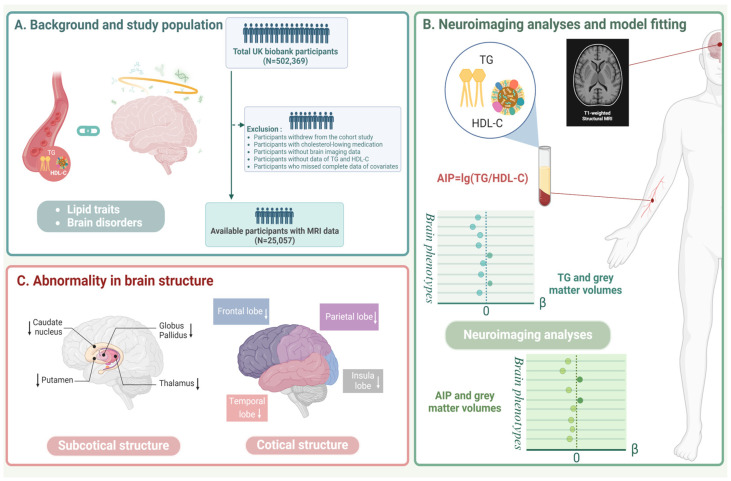
Study workflow. (**A**) To explore the effects of specific lipid traits on the brain system, we performed the investigation among 25,057 UK Biobank participants with available MRI data. Exclusion criteria were presented in panel (**A**) (right). (**B**) We conducted neuroimaging analyses to estimate the associations of TG and AIP with brain grey matter phenotypes by multiple linear regression. βs and the corresponding 95% CIs were calculated in our analyses, with TG and AIP as categorical variables and continuous variables. Subgroup analyses were performed by demographic and lifestyle factors as well. (**C**) Results demonstrated that higher TG and AIP were associated with reduced brain grey matter volumes, and details of abnormality in brain structure were depicted in panel (**C**).

Figure 1 shows the whole design of the study. A total of 25,057 individuals were included in our study, consisting of 53.5% women; participants had a mean ± SD age of 54.3 ± 7.5 years at baseline (Table 1). The median levels of TG and AIP were 1.36 mmol/L (interquartile range IQR: 1.01 mmol/L) and −0.03 (0.41), respectively. The mean ± SD lipid levels of participants were 5.8 ± 1.0 mmol/L for TC and 3.7 ± 0.8 mmol/L for LDL-C, and the mean ± SD value of HbA1c was 34.5 ± 4.3 mmol/mol. The mean ± SD levels of SBP and DBP were 134.0 ± 17.6 mmHg and 81.2 ± 10.0 mmHg, respectively. Participants with a lower level of AIP were more likely to be female and to have lower IMD or more ideal WHR. AIP levels were also lower among people without comorbidities (hypertension, diabetes, atrial fibrillation, stroke), those with healthier lifestyles (except for drinking), and lower levels of blood pressure, TC, and LDL-C. Baseline characteristics of participants by categorized TG are provided in Supplementary Table S1. After removing those with lipid-lowering drugs, a total of 8559 and 7573 prevalent cases with hypertriglyceridemia (TG levels > 1.69 mmol/L) and mixed hyperlipidemia (TG levels > 1.7 mmol/L and TC levels > 5 mmol/L) at baseline were still identified, respectively [20,21].

**Figure 2:**
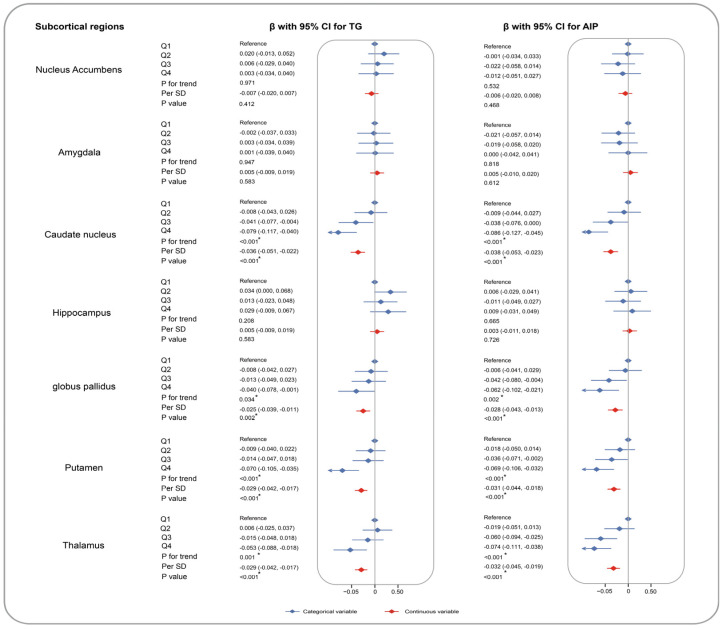
Associations of TG and AIP with brain subcortical imaging phenotypes. The brain subcortical imaging phenotypes included volumes of nucleus accumbens, amygdala, caudate nucleus, hippocampus, globus pallidus, putamen, and thalamus. We conducted the analyses to assess the associations of TG and AIP with brain subcortical structure among 25,057 participants with MRI data. Multiple linear regression models were built with adjustments for age, sex, IMD, WHR, healthy lifestyle, SBP, DBP, HbA1c, LDL-C, TC, prevalence of hypertension, diabetes, atrial fibrillation, and stroke. In our analyses, βs and corresponding 95% CIs were estimated with TG and AIP as categorical and continuous variables. The red points indicate HRs per SD, and the blue points are categorized HRs. Brain structure marked with asterisks indicates the associations of statistical significance.

**Figure 3:**
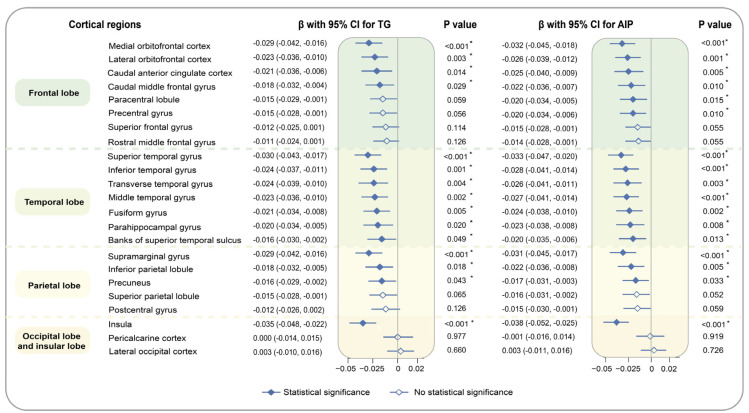
Associations of TG and AIP with brain cortical imaging phenotypes. The brain cortical imaging phenotypes consisted of frontal, temporal, parietal, occipital and insular lobes of the brain. Data from 25,057 participants were utilized to assess the associations of TG and AIP with brain cortical structure by multiple linear regression, after adjusting age, sex, IMD, WHR, healthy lifestyle, SBP, DBP, HbA1c, LDL-C, TC, prevalence of hypertension, diabetes, atrial fibrillation, and stroke. Full dots are results of significance and empty dots are those of insignificance. Brain structure marked with asterisks indicates the associations of statistical significance.

We performed multivariate linear regression analyses to assess the associations of TG and AIP with brain grey matter phenotypes after adjustment for potential covariates. The full name for each brain imaging trait is depicted in Supplementary Table S2. In terms of subcortical structure, TG and AIP, as continuous variables, showed significant inverse associations with the volumes of caudate nucleus (TG: β [95% CI] = −0.036 [−0.051, −0.022], AIP: −0.038 [−0.053, −0.023]), globus pallidus (−0.025 [−0.039, −0.011], −0.028 [−0.043, −0.013]), putamen (−0.029 [−0.042, −0.017], −0.031 [−0.044, −0.018]), and thalamus (−0.029 [−0.042, −0.017], −0.032 [−0.045, −0.019]). As for cortical regions, TG as a continuous variable is associated with reduced volumes of insula (−0.035 [−0.048, −0.022]), superior temporal gyrus (−0.03 [−0.043, −0.017]), and supramarginal gyrus (−0.029 [−0.042, −0.016]), medial orbitofrontal cortex (−0.029 [−0.042, −0.016]), etc. Based on the same significance regions as TG, those with a higher AIP level also had atrophic volumes of insula (−0.038 [−0.052, −0.025]), superior temporal gyrus (−0.033 [−0.047, −0.020]), medial orbitofrontal cortex (−0.032 [−0.045, −0.018]), and supramarginal gyrus (−0.031 [−0.045, −0.017]), etc. The findings of TG and AIP as category variables were compatible with the above results. Figure 2 and Figure 3 summarize the associations of TG and AIP as continuous variables with volumes of brain grey matter phenotypes, and results for categorical exposures and corresponding P for trends are shown in Supplementary Tables S3 and S4. 

**Figure 4:**
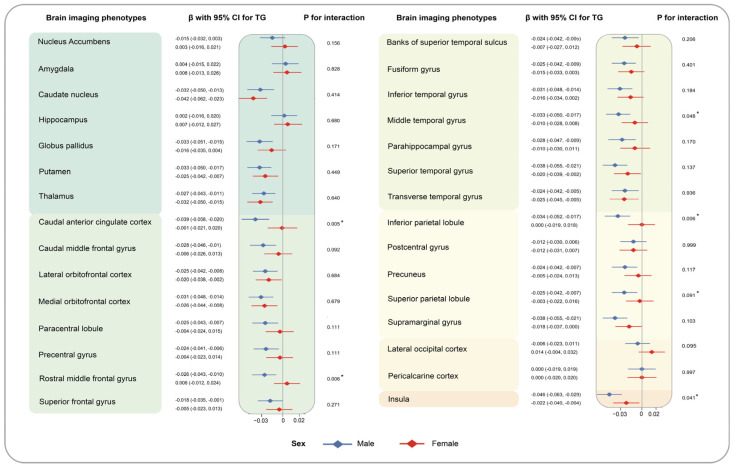
Associations of TG with brain imaging phenotypes stratified by sex. Results were presented as βs with corresponding 95% CIs with AIP as a continuous variable. P for interaction for each subgroup was calculated to evaluate comparability through the multiplicative term in the model. Brain structure marked with asterisks indicates the associations of statistical significance.

**Figure 5:**
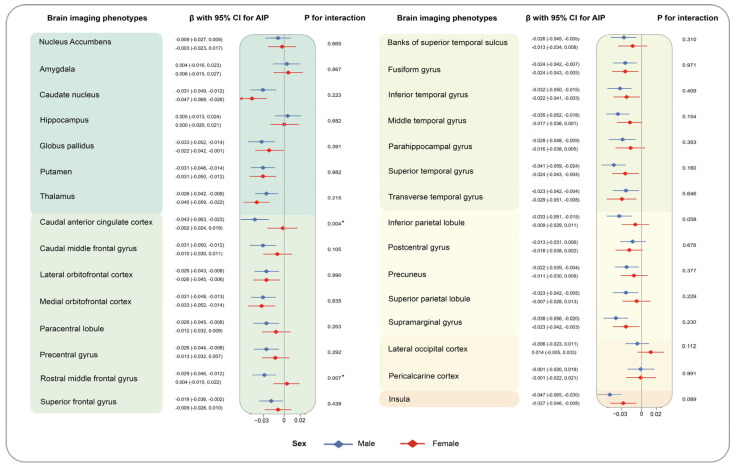
Associations of AIP with brain imaging phenotypes stratified by sex. P for interaction for each subgroup was calculated to evaluate comparability through the multiplicative term in the model. Brain structure marked with asterisks indicates the associations of statistical significance.

**Figure 6:**
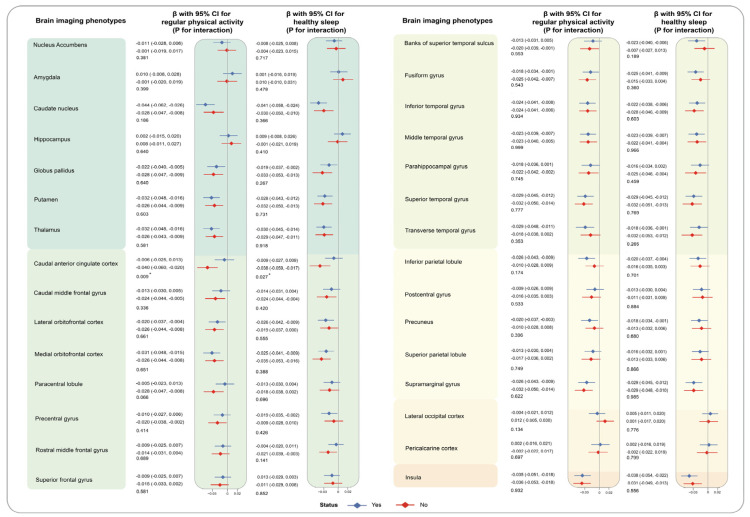
Associations of TG with brain imaging phenotypes stratified by healthy lifestyle. The analyses were stratified by regular physical activity (no/yes) and healthy sleep pattern (no/yes). βs and corresponding 95% CIs were estimated in our analyses with TG as a continuous variable. P for interaction for each subgroup was calculated to evaluate comparability through the multiplicative term in the model. Brain structure marked with asterisks indicates the associations of statistical significance.

**Figure 7:**
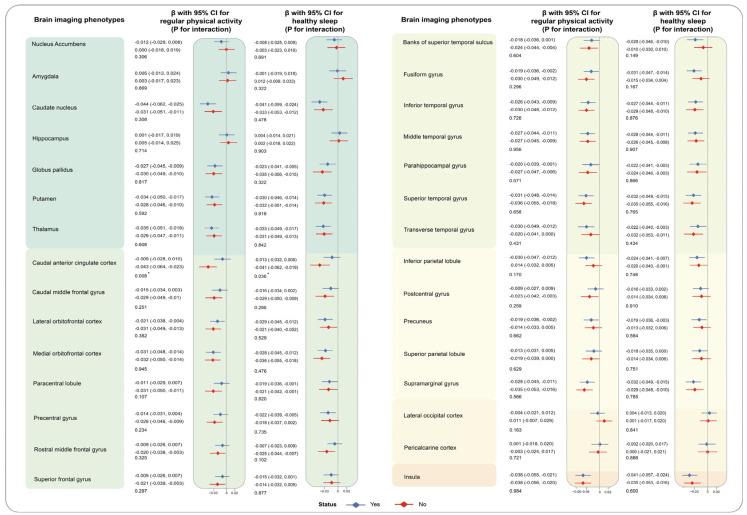
Associations of AIP with brain imaging phenotypes stratified by healthy lifestyle. The analyses were stratified by regular physical activity (no/yes) and healthy sleep pattern (no/yes). βs and corresponding 95% CIs were estimated in our analyses with AIP as a continuous variable. P for interaction for each subgroup was calculated to evaluate comparability through the multiplicative term in the model. Brain structure marked with asterisks indicates the associations of statistical significance.

Heterogeneity was observed among different genders. High levels of TG were highly related to reduced volumes of seven brain grey matter phenotypes among males (caudal anterior cingulate cortex: −0.039 [−0.058, −0.020] for male, −0.001 [−0.021, 0.020] for female, *p* for interaction = 0.005; rostral anterior cingulate cortex: −0.026 [−0.044, −0.008], 0.007 [−0.012, 0.026], *p* for interaction = 0.009; rostral middle frontal gyrus (−0.026 [−0.043, −0.010], 0.006 [−0.012, 0.024], *p* for interaction = 0.006), etc.), compared to the individuals with low level of TG. Similarly, the relationship of higher levels of AIP and decreased volumes of caudal anterior cingulate cortex(−0.043 [−0.063, −0.023] for male, −0.002 [−0.024, 0.019] for female, *p* for interaction = 0.004), rostral anterior cingulate cortex (−0.025 [−0.044, −0.007], 0.004 [−0.017, 0.024], *p* for interaction = 0.028), and rostral middle frontal gyrus (−0.029 [−0.046, −0.012], 0.004 [−0.015, 0.022], *p* for interaction = 0.007) were only detected among males but not females (Supplementary Table S5). Moreover, the synergistic effect of regular physical activity and TG on the volumes of caudal anterior cingulate cortex (yes: −0.006 [−0.025, 0.013], no: −0.040 [−0.060, −0.020], *p* for interaction = 0.009) was noticed. Similarly, we observed the more prominent association of AIP with reduced volumes of anterior cingulate cortex (−0.009 [−0.028, 0.010], −0.043 [−0.064, −0.023], *p* for interaction = 0.008). To make it more visible, results of stratified analyses by sex and regular physical activity are depicted in Figure 4, Figure 5, Figure 6 and Figure 7. The interaction effects of age, WHR, or healthy sleep pattern with TG and AIP on brain structural defects were also discovered (Figures S1 and S2 and Supplementary Tables S6–S8). In our analyses, interaction of PRS and TG hardly influences brain structural alterations (Figure S3). We also verified the robustness of conclusions by sensitivity analyses (Supplementary Tables S9–S12).

## 4. Discussion

We provided evidence based on the UK Biobank dataset that TG and AIP were adversely associated with brain grey matter volumes. Specifically, TG and AIP both correlated with atrophic grey matter volumes in four identical subcortex phenotypes, encompassing caudate nucleus, globus pallid, putamen, and thalamus. Concerning cortical structure, TG was significantly associated with reduced volumes in superior temporal gyrus, supramarginal gyrus, and insula, etc. More phenotypes affected by a higher AIP level were also observed, including medial orbitofrontal cortex, supramarginal gyrus, superior parietal lobule, and insula, etc. Additionally, we discovered the apparent sex, age, WHR, healthy sleep pattern, and regular physical activity heterogeneities in associations of TG and AIP with reduced brain grey matter volumes.

Numerous studies observed meaningful connections between lipids and neuropsychiatric diseases [22,23,24,25]. Researchers further investigated the correlations between serum lipids and cerebral β-amyloid [26], shedding new insight into the role of lipids in the pathological mechanism of neurodegenerative diseases. To explore the relevant mechanism further, some studies investigated the relationship between lipids and brain morphology [5,8,9,10,27,28]. However, existing studies either did not identify the independent lipid associations or only focused on brain regions of interest [5,8]. In a cohort study with predominantly midlife participants, considerable relationships between smaller volumes in specific brain regions and cardiovascular disease risk factors (such as lipid levels) were observed [8]. Another study found that increasing lipid levels were associated with reductions in specific brain grey matter phenotypes in youths with bipolar disorder compared with healthy controls [5]; similar results also appeared in the middle-aged and elderly population in our study.

Our investigations extend prior research by evaluating potential effects of TG and AIP on brain imaging phenotypes with a non-targeted approach. Volumes in occipital lobe seemed to be insusceptible to the serum levels of TG and AIP in our study, which is compatible with normal occipital anatomy and physiology shown in patients with schizophrenia [29]. On the contrary, it was revealed that TG contributed to reduced cortex thickness in frontal, temporal, parietal, and occipital lobes [7]. A longitudinal Atherosclerosis Risk in Communities Neurocognitive Study that included 1872 middle-aged adults reported the adverse association of TG with total brain volumes, which was consistent with our results [30]. Nonetheless, the opposite result was observed in the Dallas Heart Study with a limited sample size (1629 middle-aged population) [8]. The protective impact of HDL-C would no longer be valid at a high level [31]. All conflicting results shown in prior studies might originate from limited sample sizes, measures bias, methodological difference, and other factors such as age, sex, blood pressure, etc. For instance, no significant associations were observed in the Voxel-based morphometry analyses [28], whereas we found different results using the FreeSurfer IDPs. It was reported that there was an interactive effect between LDL-C levels and hypertension on brain structure [6]. Fortunately, with a large sample size (25,057 middle-aged and elderly adults), we could control for more potential risk factors, which enhanced the reliability of the conclusion.

A presumable mechanism through which TG and AIP could result in alterations of brain grey matter volumes is the lipid invasion model. The mechanism suggested external lipids from systemic and peripheral processes might induce neurodegeneration in the brain when the blood-brain barrier was damaged [32,33]. Specifically, invasive lipids that disrupt overall brain homeostasis would be taken by neurons, then leading to neuronal amyloid deposition in the brain [26]. Our findings provide evidence for the lipid invasion model because the cell bodies of neurons could be found in brain grey matter [34]. Another speculative mechanism underlying the association between lipids and brain structural defects is vascular pathology. AIP was approved as an indicator of insulin resistance [35] and promoted the formation of atherosclerotic LDL particles. LDL particles are more susceptible to passing through the arterial wall and binding to proteoglycans, thus making the arteries more vulnerable to oxidative modifications [36]. Hence, vascular insults and atherosclerosis were partly responsible for detrimental effects of TG and AIP on brain structure. Dyslipidemia might cause brain resting-state network disruption and CSF biomarker deposition, which is related to cognitive decline and high risks of brain disorders [37]. Considering that MRI indicates selectivity of brain changes and susceptibility for cognitive decline, our findings that TG and AIP were related to atrophic volumes of specific brain imaging phenotypes are coherent with the above mechanisms. Particularly, that the caudate nucleus, putamen, and frontal lobe are part of the mesostriatal and mesocortical pathways in the dopaminergic system are involved in cognitive and motor function regulation [38]. Putamen, the lateral and medial orbitofrontal cortexes, and insula are also known to play a crucial role in food pleasure [39]. Consequently, our findings suggested that TG and AIP led to central nervous system damage and cognitive insult by affecting brain grey matter phenotypes. Subsequently, Mendelian randomization analysis is needed to verify their causality. Further research is also warranted to evaluate lipid homeostasis in CSF due to its close connection with the brain. 

It remains to be elucidated whether associations of TG and AIP with morphological alteration of the brain differs between genders and regular physical activity. A sex interaction with men having smaller grey matter volumes was noted in our study. A higher concentration of TC was recognized as a protective factor for cognitive function among females in later life [40], which partly explained the sex difference in our results. It was acknowledged that physical activity improved cognitive function and memory, and even delayed brain aging [41]. Similarly, a more significant correlation of AIP with smaller volumes in frontal lobe appeared among participants without regular physical activity in our study. Given that adding strength training to aerobic training supplementarily affects the lipid profiles [42], different types of exercise (such as competitive sprint-trained versus endurance-trained) may affect lipid parameters in different ways. Studies to further investigate potential interaction effects between TG/AIP and types of exercise on the variation of brain structural traits are in great demand, especially in relation to aerobic exercise, strength training, and others. Moreover, further research is still needed to illuminate the specific mechanism in regard to the synergetic effect of sex or physical activity with lipid context on the alteration of brain structure.

Despite this, our study was not without limitations. First, the large sample size in the UK Biobank could output relatively credible results but the ability to generalize the results to the overall population might remain uncertain. As with other longitudinal cohort studies, there are “healthy volunteer” and ethnicity selection biases in the UK Biobank study [43]. Second, information acquired by self-report and imprecise measurement methods (measuring lipids without fasting) may bias the results. Third, although adjustments have been made, our analyses are prone to residual and unmeasured confounding. For instance, dyslipidemia is generally accompanied by abnormality in other metabolites, such as uric acid. Our analyses were performed after excluding individuals using lipid-lowering medicines, but the history of hormone use has also influenced lipid metabolism. Finally, we corrected for multiple testing by setting an FDR threshold to reduce false positive results, but it may have led us to overlook some biologically and clinically meaningful associations inversely.

## 5. Conclusions

Atrophic brain grey matter volumes with high levels of TG and AIP were observed through modeling, which provides novel clues for possible mechanisms as well as treatment and prevention strategies for brain disorders. Smaller volumes in specific phenotypes also evidenced the selectivity and susceptibility in brain response to TG and AIP, which offers scientific guidelines for region-specific therapies and prevention strategies. As modifiable factors, the discovery and application of TG and AIP have a critical and meaningful clinical value in mechanism exploration and risk prediction. These findings may improve the early diagnosis and treatment in clinical practice and contribute to prevention intervention of neuropsychiatric diseases at the population level.

## Figures and Tables

**Table 1:** Baseline characteristics of participants grouped by AIP quartiles.

Characteristic	All (N = 25,057)	Q1 ^a^ (N = 6264)	Q2 (N = 6264)	Q3 (N = 6264)	Q4 (N = 6265)
Age, year	54.3 (7.5)	53.3 (7.4)	54.3 (7.5)	54.9 (7.5)	54.6 (7.4)
Female population	13,416 (53.5%)	4754 (75.9%)	3901 (62.3%)	3012 (48.1%)	1749 (27.9%)
IMD ^b^	14.7 (12.0)	14.0 (11.5)	14.4 (11.8)	14.8 (12.2)	15.4 (12.6)
WHR ^c^					
Ideal	15,140 (60.4%)	5334 (85.2%)	4436 (70.8%)	3357 (53.6%)	2013 (32.1%)
Poor	9917 (39.6%)	930 (14.8%)	1828 (29.2%)	2907 (46.4%)	4252 (67.9%)
Lifestyle					
Never smoking	23,499 (93.8%)	5971 (95.3%)	5936 (94.8%)	5848 (93.4%)	5744 (91.7%)
Never drinking	1109 (4.4%)	214 (3.4%)	254 (4.1%)	283 (4.5%)	358 (5.7%)
Regular physical activity ^d^	13,745 (54.9%)	3823 (61.0%)	3483 (55.6%)	3331 (53.2%)	3108 (49.6%)
Healthy sleep pattern ^e^	14,822 (59.2%)	4112 (65.6%)	3859 (61.6%)	3514 (56.1%)	3337 (53.3%)
Healthy diet ^f^	10,058 (40.1%)	2884 (46.0%)	2703 (43.2%)	2359 (37.7%)	2112 (33.7%)
Cardiometabolic factors					
SBP (mmHg)	134.0 (17.6)	130.0 (17.6)	132.0 (17.7)	136.0 (17.5)	138.0 (16.5)
DBP (mmHg)	81.2 (10.0)	78.3 (9.6)	80.1 (9.7)	82.2 (9.8)	84.2 (9.6)
HbA1c (mmol/mol)	34.5 (4.3)	33.7 (3.6)	34.2 (3.7)	34.7 (4.3)	35.4 (5.3)
TC (mmol/L)	5.8 (1.0)	5.7 (1,0)	5.8 (1.0)	5.9 (1.0)	6.1 (1.0)
LDL-C (mmol/L)	3.7 (0.8)	3.3 (0.7)	3.6 (0.8)	3.8 (0.8)	3.9 (0.8)
Prevalence of comorbidities					
Hypertension	3834 (15.3%)	601 (9.6%)	835 (13.3%)	1070 (17.1%)	1328 (21.2%)
Diabetes	224 (0.9%)	31 (0.5%)	34 (0.5%)	63 (1.0%)	96 (1.5%)
Atrial fibrillation	112 (0.5%)	18 (0.3%)	31 (0.5%)	33 (0.5%)	30 (0.5%)
Stroke	76 (0.3%)	12 (0.2%)	21 (0.3%)	17 (0.3%)	26 (0.4%)

Abbreviations: index of multiple deprivation, IMD; waist-to-hip ratio, WHR; Systolic blood pressure, SBP; diastolic blood pressure, DBP; hemoglobin A1c, HbA1c; total cholesterol, TC; low-density lipoprotein cholesterol, LDL-C; atherogenic index of plasma, AIP. ^a^ AIP was categorized by quartile: Q1 (<−0.88), Q2 (−0.22 to −0.01), Q3 (−0.01 to 0.20), and Q4 (≥0.20). ^b^ IMD was measured by even domains of deprivation: crime score, community safety score, education score, employment score, health score, housing score, income score, living environment score, access to services score, and physical environment score. ^c^ WHR was calculated as waist circumference (centimeter) divided by hip circumference (centimeter) and categorized into ideal (<0.9 for men and <0.85 for women) and poor (≥0.9 for men and ≥0.85 for women). ^d^ Regular physical activity: ≥150 min of moderate-intensity per week or ≥75 min of vigorous-intensity per week, or a combination of both. ^e^ Healthy sleep pattern was measured by 5 dimensions of sleep behaviors: early chronotype, sleep 7–8 h/day, never/rarely or sometimes insomnia, no self-reported snoring, and never/rarely or sometimes excessive daytime sleepiness, and categorized into yes (≥4 healthy components) and no. ^f^ Healthy diet was measured by seven healthy diet components: Fruits: ≥3 servings/day; Vegetables: ≥3 servings/day; Fish: ≥2 servings/week; Processed meats: ≤1 serving/week; Unprocessed red meats: ≤1.5 servings/week; Whole grains: ≥3 servings/day; Refined grains: ≤1.5 servings/day, and categorized into yes (participants had ≥4 healthy diet components) and no.

## Data Availability

All the data for this study will be made available upon reasonable request to the corresponding authors.

## Supplementary Materials

The following supporting information can be downloaded at: https://www.mdpi.com/article/10.3390/nu16050672/s1, Figure S1: Associations between TG and brain imaging phenotypes by WHR; Figure S2: Associations between AIP and brain imaging phenotypes by WHR; Figure S3: Associations between TG and brain imaging phenotypes by PRS; Table S1: Baseline characteristics of participants grouped by TG quartiles; Table S2: Definitions of brain phenotypes; Table S3: Associations between TG and brain imaging phenotypes; Table S4: Associations between AIP and brain imaging phenotypes; Table S5: Associations of TG and AIP with brain imaging phenotypes by sex; Table S6: Associations of TG and AIP with brain imaging phenotypes by age; Table S7: Associations of TG and AIP with brain imaging phenotypes by WHR; Table S8: Associations of TG and AIP with brain imaging phenotypes by healthy sleep pattern and regular physical activity; Table S9: Sensitivity analysis (1–4) of the main associations between TG and brain grey matter phenotypes; Table S10: Sensitivity analysis (1–4) of the main associations between AIP and brain grey matter phenotypes; Table S11: Sensitivity analysis (5–7) of the main associations between TG and brain grey matter phenotypes; Table S12: Sensitivity analysis (5–7) of the main associations between AIP and brain grey matter phenotypes.
