# Supplementary material for: Associations of Triglycerides and Atherogenic Index of Plasma with Brain Structure in the Middle-Aged and Elderly Adults
Source: Nutrients. 2024 Feb 27;16(5):672. doi: 10.3390/nu16050672 (PMC10933770; doi:10.3390/nu16050672)
Supplement: Supplementary file 1 [file nutrients-16-00672-s001.zip › nutrients-2870430-supplementary.pdf]

Supplementary Information

Associations of Triglycerides and Atherogenic Index of Plasma with Brain Structure in the Middle-Aged and Elderly Adults

Figure S1. Associations between TG and brain imaging phenotypes by WHR 2

Figure S2. Associations between AIP and brain imaging phenotypes by WHR 3

Figure S3. Associations between TG and brain imaging phenotypes by PRS 4

Table S1. Baseline characteristics of participants grouped by TG quartiles 5

Table S2. Definitions of brain phenotypes 6

Table S3. Associations between TG and brain imaging phenotypes 7

Table S4. Associations between AIP and brain imaging phenotypes 9

Table S5. Associations of TG and AIP with brain imaging phenotypes by sex 11

Table S6. Associations of TG and AIP with brain imaging phenotypes by age 13

Table S7. Associations of TG and AIP with brain imaging phenotypes by WHR 15

Table S8. Associations of TG and AIP with brain imaging phenotypes by healthy sleep pattern and regular physical activity 17

Table S9. Sensitivity analysis (1-4) of the main associations between TG and brain grey matter phenotypes 20

Table S10. Sensitivity analysis (1-4) of the main associations between AIP and brain grey matter phenotypes 22

Table S11. Sensitivity analysis (5-7) of the main associations between TG and brain grey matter phenotypes 24

Table S12. Sensitivity analysis (5-7) of the main associations between AIP and brain grey matter phenotypes 26

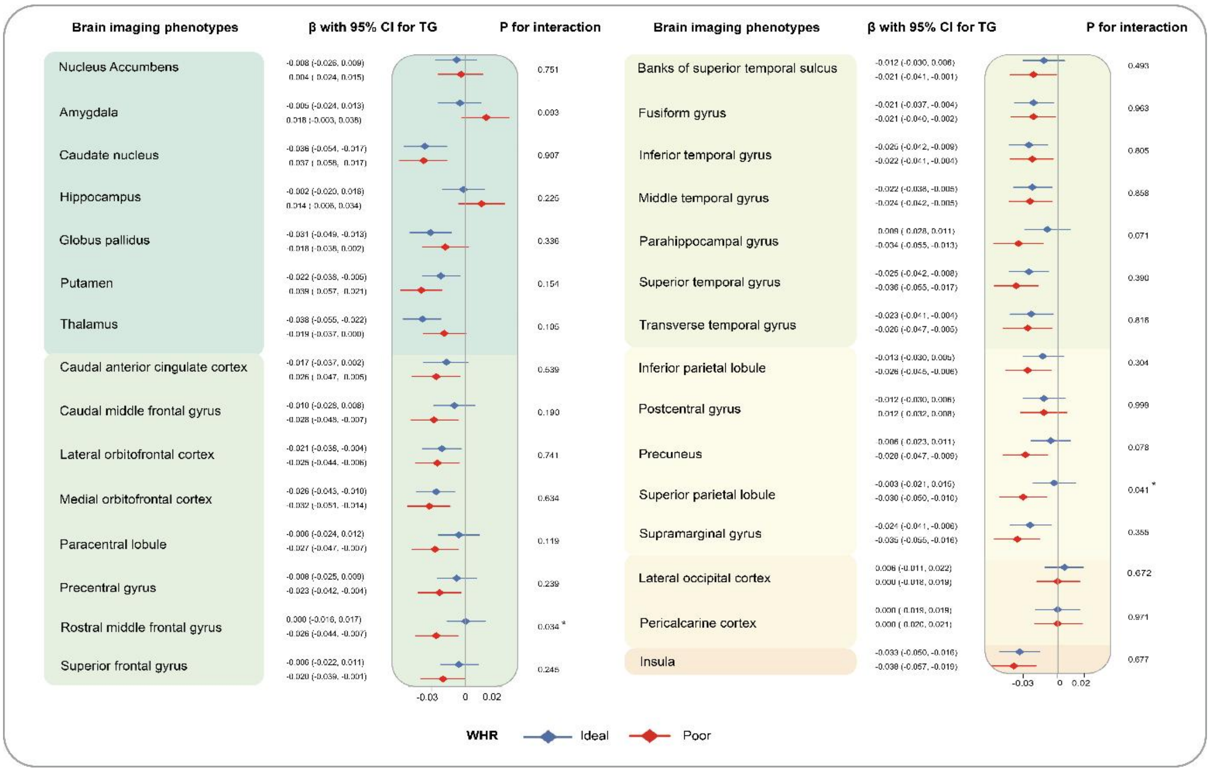

Figure S1. Associations between TG and brain imaging phenotypes by WHR

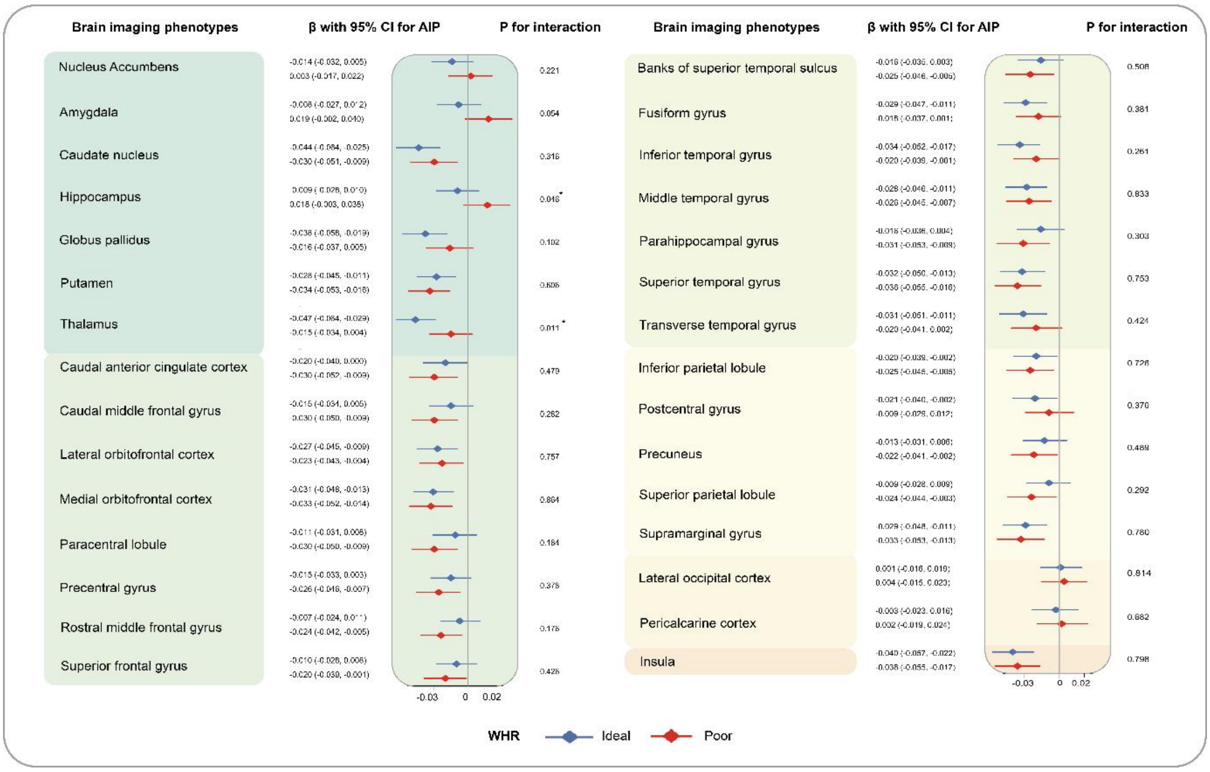

Figure S2. Associations between AIP and brain imaging phenotypes by WHR

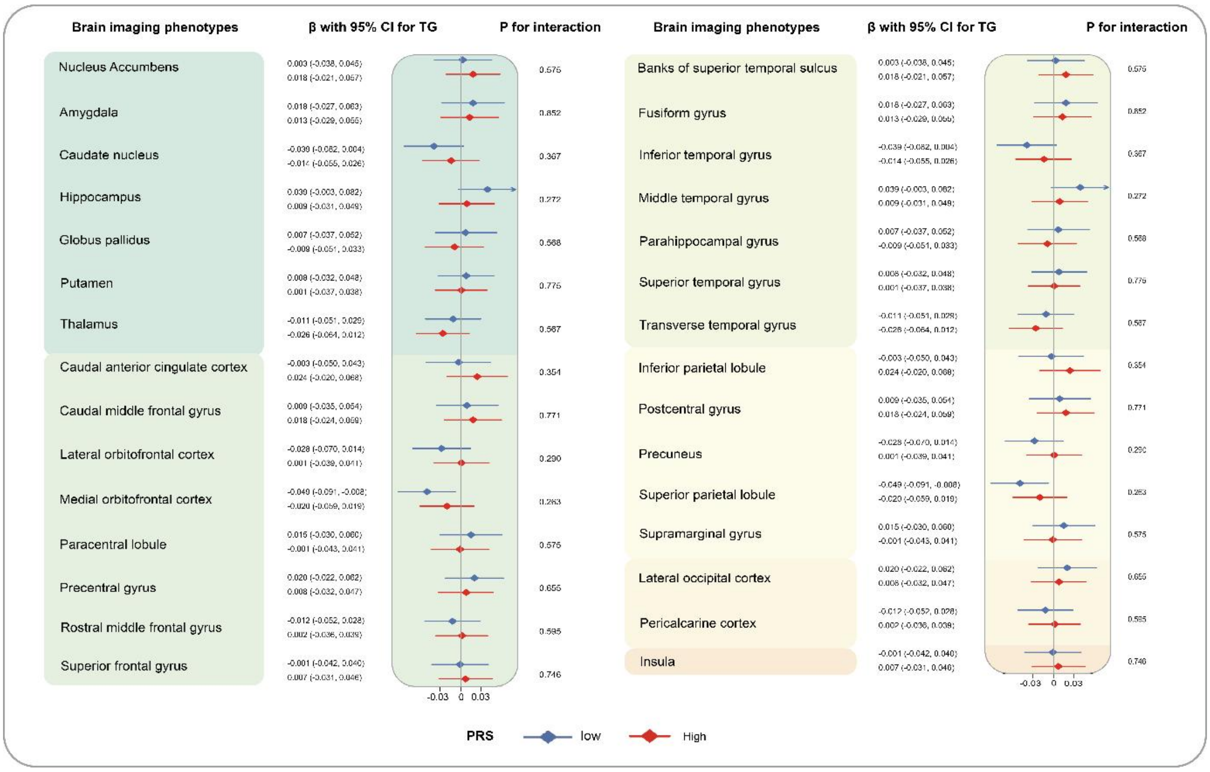

Figure S3. Associations between TG and brain imaging phenotypes by PRS

Table S1. Baseline characteristics of participants grouped by TG quartiles

Characteristic	All (N=25057)	Q1 (N=6254)	Q2 (N=6267)	Q3 (N=6271)	Q4 (N=6265)	p.overall
**Age, year**	54.3 (7.5)	52.5 (7.4)	54.4 (7.4)	55.2 (7.5)	55.0 (7.3)	<0.001
**Sex**						<0.001
Female	13416 (53.5%)	4363 (69.8%)	3747 (59.8%)	3141 (50.1%)	2165 (34.6%)	
Male	11641 (46.5%)	1891 (30.2%)	2520 (40.2%)	3130 (49.9%)	4100 (65.4%)	
**IMD**	14.7 (12.0)	14.3 (11.9)	14.3 (11.7)	15.0 (12.3)	15.1 (12.2)	<0.001
**WHR**						<0.001
ideal	15140 (60.4%)	5170 (82.7%)	4356 (69.5%)	3404 (54.3%)	2210 (35.3%)	
poor	9917 (39.6%)	1084 (17.3%)	1911 (30.5%)	2867 (45.7%)	4055 (64.7%)	
**Lifestyle**						
Never smoking	23499 (93.8%)	5955 (95.2%)	5925 (94.5%)	5857 (93.4%)	5762 (92.0%)	<0.001
Never drinking	1109 (4.4%)	262 (4.2%)	260 (4.2%)	268 (4.3%)	319 (5.1%)	0.031
Regular physical activity	13745 (54.9%)	3730 (59.6%)	3464 (55.3%)	3380 (53.9%)	3171 (50.6%)	<0.001
Healthy sleep pattern	14822 (59.2%)	4119 (65.9%)	3816 (60.9%)	3525 (56.2%)	3362 (53.7%)	<0.001
Healthy diet	10058 (40.1%)	2817 (45.0%)	2622 (41.8%)	2465 (39.3%)	2154 (34.4%)	<0.001
**Cardiometabolic factors**						
SBP	134.0 (17.6)	129.0 (17.2)	133.0 (17.5)	136.0 (17.5)	139.0 (16.9)	<0.001
DBP	81.2 (10.0)	78.2 (9.6)	80.3 (9.7)	82.1 (9.9)	84.2 (9.7)	<0.001
HbA1c	34.5 (4.3)	33.6 (3.6)	34.2 (3.8)	34.7 (4.3)	35.4 (5.2)	<0.001
TC	5.8 (1.0)	5.4 (0.9)	5.7 (10.0)	6.0 (10.0)	6.3 (1.0)	<0.001
LDL-C	3.7 (0.8)	3.2 (0.7)	3.6 (0.7)	3.82 (0.7)	4.0 (0.8)	<0.001
**Prevalence of comorbidities**						
Hypertension	3834 (15.3%)	585 (9.4%)	852 (13.6%)	1091 (17.4%)	1306 (20.8%)	<0.001
Diabetes	224 (0.9%)	34 (0.5%)	46 (0.7%)	59 (0.9%)	85 (1.4%)	<0.001
Atrial fibrillation	112 (0.5%)	26 (0.4%)	27 (0.4%)	29 (0.5%)	30 (0.5%)	0.950
Stoke	76 (0.3%)	16 (0.3%)	13 (0.2%)	20 (0.3%)	27 (0.4%)	0.122

Table S2. Definitions of brain phenotypes

brain phenotypes	Entity (Abbr)
Subcortical structure	accumbens nucleus (NAc)
	amygdala (AMYG)
	caudate nucleus (CN)
	hippocampus (HIP)
	globus pallidus (GP)
	putamen (PUT)
	thalamus (THA)
Cortical structure	
Frontal lobe	caudal anterior cingulate cortex (cACC)
	caudal middle frontal gyrus (cMFG)
	frontal pole (FP)
	lateral orbitofrontal cortex (IOFC)
	medial orbitofrontal cortex (mOFC)
	paracentral lobule (PCL)
	pars opercularis (OP)
	pars orbitalis (ORB)
	pars triangularis (TRI)
	precentral gyrus (PCG)
	rostral anterior cingulate cortex (rACC)
	rostral middle frontal gyrus (RMFG)
	superior frontal gyrus (SFG)
Temporal lobe	banks of superior temporal sulcus (Bankssts)
	entorhinal cortex (EC)
	fusiform gyrus (FG)
	inferior temporal gyrus (ITG)
	middle temporal gyrus (MTG)
	parahippocampal gyrus (PHG)
	superior temporal gyrus (STG)
	transverse temporal gyrus (TTG)
Parietal lobe	inferior parietal lobule (IPL)
	isthmus cingulate (Isthmus)
	postcentral gyrus (PoCG)
	posterior cingulate cortex (PCC)
	precuneus (PCUN)
	superior parietal lobule (SPL)
	supramarginal gyrus (SMG)
Occipital lobe	cuneus (CUN)
	lateral occipital cortex (LOC)
	lingual gyrus (LG)
	pericalcarine cortex (PCAL)
Insula lobe	insula (INS)

Table S3. Associations between TG and brain imaging phenotypes

Brain stucture[a]	TG[b]				*P* for trend	*β* per IQR[c]	*P* value FDR[c]
	Q1	Q2	Q3	Q4			
**Volume of subcortical structure**							
NAc	Reference	0.020 (-0.013, 0.052)	0.006 (-0.029, 0.040)	0.003 (-0.034, 0.040)	0.971	-0.007 (-0.020, 0.007)	0.412
AMYG	Reference	-0.002 (-0.037, 0.033)	0.003 (-0.034, 0.039)	0.001 (-0.039, 0.040)	0.947	0.005 (-0.009, 0.019)	0.583
CN	Reference	-0.008 (-0.043, 0.026)	-0.041 (-0.077, -0.004)	-0.079 (-0.117, -0.040)	<0.001	-0.036 (-0.051, -0.022)	<0.001
HIP	Reference	0.034 (0.000, 0.068)	0.013 (-0.023, 0.048)	0.029 (-0.009, 0.067)	0.208	0.005 (-0.009, 0.019)	0.583
GP	Reference	-0.008 (-0.042, 0.027)	-0.013 (-0.049, 0.023)	-0.040 (-0.078, -0.001)	0.034	-0.025 (-0.039, -0.011)	0.002
PUT	Reference	-0.009 (-0.040, 0.022)	-0.014 (-0.047, 0.018)	-0.070 (-0.105, -0.035)	<0.001	-0.029 (-0.042, -0.017)	<0.001
THA	Reference	0.006 (-0.025, 0.037)	-0.015 (-0.048, 0.018)	-0.053 (-0.088, -0.018)	0.001	-0.029 (-0.042, -0.017)	<0.001
**Volume of cortical structure**							
**Frontal lobe**							
cACC	Reference	-0.007 (-0.043, 0.030)	0.000 (-0.038, 0.038)	-0.055 (-0.096, -0.014)	0.008	-0.021 (-0.036, -0.006)	0.014
cMFG	Reference	0.020 (-0.015, 0.054)	-0.004 (-0.040, 0.033)	-0.031 (-0.070, 0.007)	0.065	-0.018 (-0.032, -0.004)	0.029
FP	Reference	0.016 (-0.019, 0.051)	0.033 (-0.003, 0.070)	0.054 (0.015, 0.094)	0.005	0.020 (0.005, 0.034)	0.018
lOFC	Reference	0.003 (-0.029, 0.035)	-0.004 (-0.038, 0.030)	-0.041 (-0.077, -0.005)	0.020	-0.023 (-0.036, -0.010)	0.003
mOFC	Reference	-0.019 (-0.050, 0.013)	-0.029 (-0.062, 0.005)	-0.074 (-0.109, -0.038)	<0.001	-0.029 (-0.042, -0.016)	<0.001
PCL	Reference	0.024 (-0.010, 0.058)	0.009 (-0.027, 0.045)	-0.014 (-0.053, 0.024)	0.361	-0.015 (-0.029, -0.001)	0.059
OP	Reference	0.003 (-0.031, 0.037)	0.010 (-0.026, 0.046)	-0.012 (-0.050, 0.027)	0.543	-0.009 (-0.023, 0.005)	0.276
ORB	Reference	0.010 (-0.022, 0.043)	0.031 (-0.004, 0.065)	-0.012 (-0.049, 0.025)	0.548	-0.008 (-0.021, 0.006)	0.333
TRI	Reference	0.014 (-0.020, 0.047)	0.022 (-0.014, 0.057)	-0.007 (-0.045, 0.031)	0.654	-0.007 (-0.021, 0.006)	0.374
PCG	Reference	0.033 (0.000, 0.066)	0.017 (-0.017, 0.051)	-0.010 (-0.047, 0.027)	0.429	-0.015 (-0.028, -0.001)	0.056
rACC	Reference	0.013 (-0.021, 0.047)	0.000 (-0.035, 0.036)	-0.019 (-0.058, 0.019)	0.273	-0.011 (-0.024, 0.003)	0.189
RMFG	Reference	-0.001 (-0.033, 0.030)	0.005 (-0.028, 0.038)	-0.020 (-0.055, 0.015)	0.274	-0.011 (-0.024, 0.001)	0.127
SFG	Reference	0.015 (-0.016, 0.047)	0.001 (-0.032, 0.035)	-0.012 (-0.048, 0.023)	0.428	-0.012 (-0.025, 0.001)	0.114
**Temporal lobe**							
Bankssts	Reference	-0.001 (-0.036, 0.033)	-0.007 (-0.043, 0.029)	-0.015 (-0.054, 0.023)	0.442	-0.016 (-0.030, -0.002)	0.049
EC	Reference	-0.002 (-0.037, 0.034)	-0.010 (-0.047, 0.027)	-0.011 (-0.051, 0.029)	0.593	-0.004 (-0.018, 0.011)	0.660
FG	Reference	0.001 (-0.031, 0.033)	-0.011 (-0.045, 0.022)	-0.035 (-0.071, 0.001)	0.045	-0.021 (-0.034, -0.008)	0.005
ITG	Reference	-0.002 (-0.033, 0.030)	-0.017 (-0.050, 0.017)	-0.051 (-0.087, -0.015)	0.004	-0.024 (-0.037, -0.011)	0.001
MTG	Reference	-0.013 (-0.045, 0.018)	-0.019 (-0.052, 0.015)	-0.044 (-0.080, -0.009)	0.014	-0.023 (-0.036, -0.010)	0.002
PHG	Reference	0.008 (-0.028, 0.044)	-0.009 (-0.047, 0.029)	-0.035 (-0.075, 0.006)	0.063	-0.020 (-0.034, -0.005)	0.020
STG	Reference	-0.024 (-0.057, 0.008)	-0.018 (-0.052, 0.016)	-0.071 (-0.108, -0.035)	<0.001	-0.030 (-0.043, -0.017)	<0.001

TTG	Reference	-0.001 (-0.037, 0.034)	-0.017 (-0.055, 0.020)	-0.053 (-0.093, -0.013)	0.006	-0.024 (-0.039, -0.010)	0.004
**Parietal lobe**							
IPL	Reference	0.007 (-0.026, 0.040)	-0.007 (-0.041, 0.028)	-0.027 (-0.064, 0.011)	0.126	-0.018 (-0.032, -0.005)	0.018
isthmus cingulate	Reference	0.011 (-0.022, 0.044)	0.008 (-0.027, 0.042)	-0.030 (-0.068, 0.007)	0.073	-0.012 (-0.026, 0.001)	0.115
PoCG	Reference	0.016 (-0.018, 0.050)	0.015 (-0.020, 0.050)	-0.015 (-0.053, 0.023)	0.378	-0.012 (-0.026, 0.002)	0.126
PCC	Reference	0.009 (-0.024, 0.043)	0.007 (-0.029, 0.042)	-0.022 (-0.060, 0.016)	0.228	-0.010 (-0.024, 0.003)	0.189
PCUN	Reference	0.018 (-0.014, 0.051)	0.010 (-0.024, 0.044)	-0.017 (-0.054, 0.019)	0.264	-0.016 (-0.029, -0.002)	0.043
SPL	Reference	0.025 (-0.008, 0.059)	0.028 (-0.008, 0.063)	-0.010 (-0.048, 0.028)	0.479	-0.015 (-0.028, -0.001)	0.065
SMG	Reference	-0.017 (-0.050, 0.016)	-0.016 (-0.050, 0.018)	-0.061 (-0.097, -0.024)	0.001	-0.029 (-0.042, -0.016)	<0.001
**Occipital lobe**							
CUN	Reference	0.017 (-0.017, 0.051)	0.041 (0.005, 0.076)	0.013 (-0.025, 0.052)	0.514	0.003 (-0.011, 0.017)	0.675
LOC	Reference	0.028 (-0.004, 0.060)	0.030 (-0.003, 0.064)	0.020 (-0.015, 0.056)	0.325	0.003 (-0.010, 0.016)	0.660
LG	Reference	0.016 (-0.019, 0.051)	0.015 (-0.022, 0.051)	0.002 (-0.037, 0.041)	0.998	-0.004 (-0.019, 0.010)	0.614
PCAL	Reference	0.004 (-0.031, 0.040)	0.017 (-0.020, 0.055)	0.006 (-0.034, 0.046)	0.781	0.000 (-0.014, 0.015)	0.977
**Insula lobe**							
INS	Reference	-0.018 (-0.050, 0.014)	-0.036 (-0.069, -0.002)	-0.080 (-0.115, -0.044)	<0.001	-0.035 (-0.048, -0.022)	<0.001

Abbreviations: IQR, interquartile range; FDR, false discovery rate.

a Brain structures included subcortex and cortex. The abbreviations of the phenotypes were shown in Table S1.

b TG was categorized into four groups according to quartiles: Q1 (<0.97 mmol/L), Q2 (0.97 to 1.36 mmol/L), Q3 (1.36 to 1.98 mmol/L), Q4 (≥1.98 mmol/L).

c The '*P* value FDR' is a measure of the error detection rate obtained by correcting for the difference significance *P* value. A low '*P* value FDR value' (typically less than 0.05) suggests statistical significance.

Table S4. Associations between AIP and brain imaging phenotypes

Brain stucture[a]	AIP[b]				*P* for trend	*β* per IQR[c]	*P* value FDR[c]
	Q1	Q2	Q3	Q4			
**Volume of subcortical structure**							
NAc	Reference	-0.001 (-0.034, 0.033)	-0.022 (-0.058, 0.014)	-0.012 (-0.051, 0.027)	0.532	-0.006 (-0.020, 0.008)	0.468
AMYG	Reference	-0.021 (-0.057, 0.014)	-0.019 (-0.058, 0.020)	0.000 (-0.042, 0.041)	0.818	0.005 (-0.010, 0.020)	0.612
CN	Reference	-0.009 (-0.044, 0.027)	-0.038 (-0.076, 0.000)	-0.086 (-0.127, -0.045)	<0.001	-0.038 (-0.053, -0.023)	<0.001
HIP	Reference	0.006 (-0.029, 0.041)	-0.011 (-0.049, 0.027)	0.009 (-0.031, 0.049)	0.665	0.003 (-0.011, 0.018)	0.726
GP	Reference	-0.006 (-0.041, 0.029)	-0.042 (-0.080, -0.004)	-0.062 (-0.102, -0.021)	0.002	-0.028 (-0.043, -0.013)	0.001
PUT	Reference	-0.018 (-0.050, 0.014)	-0.036 (-0.071, -0.002)	-0.069 (-0.106, -0.032)	<0.001	-0.031 (-0.044, -0.018)	<0.001
THA	Reference	-0.019 (-0.051, 0.013)	-0.060 (-0.094, -0.025)	-0.074 (-0.111, -0.038)	<0.001	-0.032 (-0.045, -0.019)	<0.001
**Volume of cortical structure**							
**Frontal lobe**							
cACC	Reference	0.001 (-0.036, 0.038)	-0.019 (-0.059, 0.021)	-0.064 (-0.106, -0.021)	0.001	-0.025 (-0.040, -0.009)	0.005
cMFG	Reference	0.017 (-0.018, 0.052)	-0.022 (-0.061, 0.016)	-0.034 (-0.075, 0.007)	0.050	-0.022 (-0.036, -0.007)	0.010
FP	Reference	0.021 (-0.015, 0.057)	0.048 (0.009, 0.086)	0.048 (0.007, 0.089)	0.025	0.021 (0.007, 0.036)	0.010
lOFC	Reference	0.000 (-0.033, 0.033)	-0.025 (-0.061, 0.010)	-0.059 (-0.098, -0.021)	0.001	-0.026 (-0.039, -0.012)	0.001
mOFC	Reference	-0.014 (-0.047, 0.018)	-0.043 (-0.078, -0.008)	-0.081 (-0.119, -0.044)	<0.001	-0.032 (-0.045, -0.018)	<0.001
PCL	Reference	0.029 (-0.006, 0.064)	-0.003 (-0.041, 0.035)	-0.021 (-0.061, 0.020)	0.160	-0.020 (-0.034, -0.005)	0.015
OP	Reference	-0.010 (-0.045, 0.025)	-0.008 (-0.046, 0.030)	-0.026 (-0.066, 0.015)	0.203	-0.010 (-0.025, 0.004)	0.210
ORB	Reference	0.011 (-0.023, 0.045)	0.002 (-0.035, 0.038)	-0.025 (-0.064, 0.013)	0.120	-0.010 (-0.024, 0.004)	0.204
TRI	Reference	0.006 (-0.029, 0.040)	-0.003 (-0.040, 0.034)	-0.020 (-0.059, 0.020)	0.265	-0.010 (-0.024, 0.004)	0.210
PCG	Reference	0.026 (-0.008, 0.059)	-0.016 (-0.052, 0.020)	-0.029 (-0.068, 0.009)	0.058	-0.020 (-0.034, -0.006)	0.010
rACC	Reference	0.013 (-0.022, 0.048)	-0.007 (-0.045, 0.031)	-0.023 (-0.064, 0.017)	0.170	-0.012 (-0.027, 0.002)	0.136
RMFG	Reference	0.011 (-0.021, 0.043)	-0.005 (-0.040, 0.029)	-0.026 (-0.063, 0.011)	0.101	-0.014 (-0.028, -0.001)	0.055
SFG	Reference	0.018 (-0.014, 0.050)	-0.029 (-0.064, 0.006)	-0.023 (-0.060, 0.015)	0.137	-0.015 (-0.028, -0.001)	0.055
**Temporal lobe**							
Bankssts	Reference	0.013 (-0.037, 0.063)	-0.010 (-0.061, 0.041)	-0.048 (-0.100, 0.003)	0.010	-0.022 (-0.039, -0.005)	0.020
EC	Reference	0.027 (-0.026, 0.080)	0.004 (-0.049, 0.058)	-0.016 (-0.069, 0.038)	0.233	-0.008 (-0.027, 0.010)	0.421
FG	Reference	-0.016 (-0.063, 0.031)	-0.036 (-0.084, 0.012)	-0.063 (-0.111, -0.015)	0.004	-0.023 (-0.039, -0.007)	0.011
ITG	Reference	-0.035 (-0.082, 0.012)	-0.066 (-0.113, -0.019)	-0.105 (-0.153, -0.057)	<0.001	-0.034 (-0.050, -0.018)	<0.001
MTG	Reference	-0.008 (-0.054, 0.039)	-0.037 (-0.084, 0.010)	-0.077 (-0.124, -0.030)	<0.001	-0.032 (-0.048, -0.016)	<0.001
PHG	Reference	0.027 (-0.025, 0.078)	-0.032 (-0.084, 0.021)	-0.073 (-0.126, -0.021)	<0.001	-0.036 (-0.053, -0.018)	<0.001
STG	Reference	-0.005 (-0.053, 0.042)	-0.031 (-0.079, 0.017)	-0.080 (-0.129, -0.032)	<0.001	-0.032 (-0.048, -0.016)	<0.001

TTG	Reference	0.021 (-0.030, 0.073)	-0.020 (-0.072, 0.033)	-0.045 (-0.098, 0.008)	0.016	-0.023 (-0.041, -0.005)	0.020
**Parietal lobe**							
IPL	Reference	0.018 (-0.016, 0.052)	-0.018 (-0.055, 0.018)	-0.048 (-0.087, -0.009)	0.005	-0.022 (-0.036, -0.008)	0.005
isthmus cingulate	Reference	0.006 (-0.028, 0.040)	-0.014 (-0.050, 0.023)	-0.034 (-0.073, 0.006)	0.056	-0.014 (-0.029, 0.000)	0.064
PoCG	Reference	0.011 (-0.023, 0.046)	-0.020 (-0.057, 0.018)	-0.030 (-0.070, 0.010)	0.083	-0.015 (-0.030, -0.001)	0.059
PCC	Reference	0.029 (-0.005, 0.064)	-0.005 (-0.042, 0.032)	-0.018 (-0.058, 0.022)	0.191	-0.015 (-0.029, 0.000)	0.064
PCUN	Reference	0.000 (-0.033, 0.033)	-0.019 (-0.055, 0.017)	-0.037 (-0.075, 0.002)	0.042	-0.017 (-0.031, -0.003)	0.033
SPL	Reference	0.008 (-0.027, 0.043)	0.002 (-0.036, 0.039)	-0.031 (-0.072, 0.009)	0.079	-0.016 (-0.031, -0.002)	0.052
SMG	Reference	-0.004 (-0.037, 0.029)	-0.037 (-0.073, -0.001)	-0.066 (-0.105, -0.027)	<0.001	-0.031 (-0.045, -0.017)	<0.001
**Occipital lobe**							
CUN	Reference	0.009 (-0.026, 0.044)	0.025 (-0.013, 0.063)	0.002 (-0.038, 0.043)	0.999	0.002 (-0.012, 0.017)	0.782
LOC	Reference	0.019 (-0.013, 0.051)	0.031 (-0.004, 0.066)	0.011 (-0.026, 0.049)	0.700	0.003 (-0.011, 0.016)	0.726
LG	Reference	0.010 (-0.026, 0.046)	0.013 (-0.025, 0.052)	-0.001 (-0.042, 0.040)	0.856	-0.006 (-0.021, 0.009)	0.524
PCAL	Reference	-0.001 (-0.037, 0.035)	0.014 (-0.025, 0.054)	0.002 (-0.040, 0.044)	0.899	-0.001 (-0.016, 0.014)	0.920
**Insula lobe**							
INS	Reference	-0.010 (-0.043, 0.022)	-0.052 (-0.087, -0.017)	-0.088 (-0.125, -0.050)	<0.001	-0.038 (-0.052, -0.025)	<0.001

Abbreviations: IQR, interquartile range; FDR, false discovery rate.

a Brain structures included subcortex and cortex. The abbreviations of the phenotypes were shown in Table S1.

b AIP was categorized into four groups according to quartiles: Q1 (<-0.88), Q2 (-0.22 to -0.01), Q3 (-0.01 to 0.20) and Q4 (≥0.20).

c The '*P* value FDR' is a measure of the error detection rate obtained by correcting for the difference significance *P* value. A low '*P* value FDR value' (typically less than 0.05) suggests statistical significance.

Table S5. Associations of TG and AIP with brain imaging phenotypes by sex

Brain structures	TG		*P* for interaction	AIP		*P* for interaction
	Male	Female		Male	Female	
	β per IQR	*β* per IQR		*β* per IQR	*β* per IQR	
**Volume of subcortical structure**						
NAc	-0.015 (-0.032, 0.003)	0.003 (-0.016, 0.021)	0.156	-0.009 (-0.027, 0.009)	-0.003 (-0.023, 0.017)	0.665
AMYG	0.004 (-0.015, 0.022)	0.006 (-0.013, 0.026)	0.828	0.004 (-0.016, 0.023)	0.006 (-0.015, 0.027)	0.867
CN	-0.032 (-0.050, -0.013)	-0.042 (-0.062, -0.023)	0.414	-0.031 (-0.049, -0.012)	-0.047 (-0.068, -0.026)	0.223
HIP	0.002 (-0.016, 0.020)	0.007 (-0.012, 0.027)	0.680	0.005 (-0.013, 0.024)	0.000 (-0.020, 0.021)	0.682
GP	-0.033 (-0.051, -0.015)	-0.016 (-0.035, 0.004)	0.171	-0.033 (-0.052, -0.014)	-0.022 (-0.042, -0.001)	0.391
PUT	-0.033 (-0.050, -0.017)	-0.025 (-0.042, -0.007)	0.449	-0.031 (-0.048, -0.014)	-0.031 (-0.050, -0.012)	0.982
THA	-0.027 (-0.043, -0.011)	-0.032 (-0.050, -0.015)	0.640	-0.026 (-0.042, -0.009)	-0.040 (-0.059, -0.022)	0.215
**Volume of cortical structure**						
**Frontal lobe**						
cACC	-0.039 (-0.058, -0.020)	-0.001 (-0.021, 0.020)	0.005	-0.043 (-0.063, -0.023)	-0.002 (-0.024, 0.019)	0.004
cMFG	-0.028 (-0.046, -0.010)	-0.006 (-0.026, 0.013)	0.092	-0.031 (-0.050, -0.012)	-0.010 (-0.030, 0.011)	0.105
FP	0.010 (-0.008, 0.029)	0.030 (0.011, 0.050)	0.122	0.014 (-0.005, 0.033)	0.030 (0.009, 0.051)	0.246
lOFC	-0.025 (-0.042, -0.008)	-0.020 (-0.038, -0.002)	0.684	-0.026 (-0.043, -0.008)	-0.026 (-0.045, -0.006)	0.990
mOFC	-0.031 (-0.048, -0.014)	-0.026 (-0.044, -0.008)	0.679	-0.031 (-0.048, -0.013)	-0.033 (-0.052, -0.014)	0.835
PCL	-0.025 (-0.043, -0.007)	-0.004 (-0.024, 0.015)	0.111	-0.026 (-0.045, -0.008)	-0.012 (-0.032, 0.009)	0.263
OP	-0.009 (-0.028, 0.009)	-0.008 (-0.028, 0.011)	0.932	-0.006 (-0.025, 0.012)	-0.015 (-0.036, 0.005)	0.497
ORB	-0.011 (-0.029, 0.006)	-0.004 (-0.022, 0.015)	0.532	-0.012 (-0.030, 0.006)	-0.008 (-0.027, 0.012)	0.704
TRI	-0.015 (-0.033, 0.002)	0.002 (-0.017, 0.021)	0.160	-0.015 (-0.033, 0.003)	-0.004 (-0.024, 0.016)	0.374
PCG	-0.024 (-0.041, -0.006)	-0.004 (-0.023, 0.014)	0.111	-0.026 (-0.044, -0.008)	-0.013 (-0.032, 0.007)	0.292
rACC	-0.026 (-0.044, -0.008)	0.007 (-0.012, 0.026)	0.009	-0.025 (-0.044, -0.007)	0.004 (-0.017, 0.024)	0.028
RMFG	-0.026 (-0.043, -0.010)	0.006 (-0.012, 0.024)	0.006	-0.029 (-0.046, -0.012)	0.004 (-0.015, 0.022)	0.007
SFG	-0.018 (-0.035, -0.001)	-0.005 (-0.023, 0.013)	0.271	-0.019 (-0.036, -0.002)	-0.009 (-0.028, 0.010)	0.439
**Temporal lobe**						
Bankssts	-0.024 (-0.042, -0.005)	-0.007 (-0.027, 0.012)	0.206	-0.026 (-0.045, -0.008)	-0.013 (-0.034, 0.008)	0.310
EC	-0.007 (-0.026, 0.012)	0.001 (-0.020, 0.021)	0.560	-0.009 (-0.028, 0.011)	-0.002 (-0.023, 0.019)	0.623
FG	-0.025 (-0.042, -0.009)	-0.015 (-0.033, 0.003)	0.401	-0.024 (-0.042, -0.007)	-0.024 (-0.043, -0.005)	0.971
ITG	-0.031 (-0.048, -0.014)	-0.016 (-0.034, 0.002)	0.184	-0.032 (-0.050, -0.015)	-0.022 (-0.041, -0.003)	0.409
MTG	-0.033 (-0.050, -0.017)	-0.010 (-0.028, 0.008)	0.048	-0.035 (-0.052, -0.018)	-0.017 (-0.036, 0.001)	0.154
PHG	-0.028 (-0.047, -0.009)	-0.010 (-0.030, 0.011)	0.170	-0.028 (-0.048, -0.009)	-0.016 (-0.038, 0.005)	0.393

STG	-0.038 (-0.055, -0.021)	-0.020 (-0.039, -0.002)	0.137	-0.041 (-0.059, -0.024)	-0.024 (-0.043, -0.004)	0.160
TTG	-0.024 (-0.042, -0.005)	-0.025 (-0.045, -0.005)	0.936	-0.023 (-0.042, -0.004)	-0.029 (-0.051, -0.008)	0.646
**Parietal lobe**						
IPL	-0.034 (-0.052, -0.017)	0.000 (-0.019, 0.018)	0.006	-0.033 (-0.051, -0.015)	-0.009 (-0.029, 0.011)	0.058
isthmus cingulate	-0.023 (-0.041, -0.005)	0.000 (-0.019, 0.019)	0.063	-0.022 (-0.040, -0.004)	-0.005 (-0.025, 0.014)	0.209
PoCG	-0.012 (-0.030, 0.006)	-0.012 (-0.031, 0.007)	0.999	-0.013 (-0.031, 0.006)	-0.018 (-0.038, 0.002)	0.678
PCC	-0.028 (-0.046, -0.010)	0.010 (-0.009, 0.029)	0.002	-0.030 (-0.049, -0.012)	0.005 (-0.016, 0.025)	0.007
PCUN	-0.024 (-0.042, -0.007)	-0.005 (-0.024, 0.013)	0.117	-0.022 (-0.039, -0.004)	-0.011 (-0.030, 0.009)	0.377
SPL	-0.025 (-0.042, -0.007)	-0.003 (-0.022, 0.016)	0.091	-0.023 (-0.042, -0.005)	-0.007 (-0.028, 0.013)	0.229
SMG	-0.038 (-0.055, -0.021)	-0.018 (-0.037, 0.000)	0.103	-0.038 (-0.056, -0.020)	-0.023 (-0.042, -0.003)	0.230
**Occipital lobe**						
CUN	0.002 (-0.016, 0.020)	0.005 (-0.015, 0.024)	0.812	-0.001 (-0.020, 0.018)	0.006 (-0.014, 0.027)	0.573
LOC	-0.006 (-0.023, 0.011)	0.014 (-0.004, 0.032)	0.095	-0.006 (-0.023, 0.011)	0.014 (-0.005, 0.033)	0.112
LG	-0.014 (-0.032, 0.005)	0.006 (-0.013, 0.026)	0.126	-0.012 (-0.031, 0.007)	0.002 (-0.019, 0.023)	0.284
PCAL	0.000 (-0.019, 0.019)	0.000 (-0.020, 0.020)	0.997	-0.001 (-0.020, 0.019)	-0.001 (-0.022, 0.021)	0.991
**Insula lobe**						
INS	-0.046 (-0.063, -0.029)	-0.022 (-0.040, -0.004)	0.041	-0.047 (-0.065, -0.030)	-0.027 (-0.046, -0.008)	0.089

Abbreviations of the phenotype were shown in Table S1.

Table S6. Associations of TG and AIP with brain imaging phenotypes by age

Brain structures	TG		*P* for interaction	AIP		*P* for interaction
	< 65	≥ 65		< 65	≥ 65	
	β per IQR	*β* per IQR		*β* per IQR	*β* per IQR	
**Volume of subcortical structure**						
NAc	-0.007 (-0.023, 0.008)	0.009 (-0.018, 0.035)	0.276	0.003 (-0.013, 0.018)	-0.006 (-0.033, 0.021)	0.554
AMYG	0.011 (-0.005, 0.027)	-0.016 (-0.043, 0.012)	0.086	0.013 (-0.004, 0.029)	-0.022 (-0.049, 0.006)	0.023
CN	-0.030 (-0.046, -0.014)	-0.054 (-0.082, -0.027)	0.111	-0.027 (-0.044, -0.011)	-0.064 (-0.091, -0.036)	0.016
HIP	0.009 (-0.006, 0.025)	-0.004 (-0.031, 0.023)	0.369	0.014 (-0.002, 0.031)	-0.016 (-0.043, 0.011)	0.038
GP	-0.024 (-0.040, -0.009)	-0.024 (-0.051, 0.003)	0.999	-0.022 (-0.038, -0.006)	-0.038 (-0.065, -0.011)	0.268
PUT	-0.022 (-0.036, -0.008)	-0.045 (-0.070, -0.020)	0.106	-0.017 (-0.032, -0.002)	-0.056 (-0.081, -0.031)	0.004
THA	-0.028 (-0.042, -0.013)	-0.025 (-0.050, 0.000)	0.848	-0.022 (-0.036, -0.007)	-0.042 (-0.067, -0.017)	0.136
**Volume of cortical structure**						
**Frontal lobe**						
cACC	-0.018 (-0.034, -0.001)	-0.029 (-0.057, 0.000)	0.485	-0.018 (-0.035, -0.001)	-0.037 (-0.065, -0.008)	0.243
cMFG	-0.017 (-0.033, -0.001)	-0.014 (-0.042, 0.013)	0.865	-0.016 (-0.032, 0.001)	-0.027 (-0.054, 0.000)	0.443
FP	0.026 (0.010, 0.042)	0.004 (-0.024, 0.031)	0.150	0.035 (0.018, 0.051)	-0.010 (-0.038, 0.017)	0.003
lOFC	-0.018 (-0.033, -0.003)	-0.032 (-0.058, -0.007)	0.321	-0.015 (-0.030, 0.000)	-0.046 (-0.072, -0.021)	0.026
mOFC	-0.023 (-0.037, -0.008)	-0.044 (-0.069, -0.019)	0.137	-0.018 (-0.033, -0.003)	-0.062 (-0.087, -0.036)	0.002
PCL	-0.011 (-0.026, 0.005)	-0.024 (-0.051, 0.003)	0.380	-0.011 (-0.027, 0.006)	-0.035 (-0.063, -0.008)	0.099
OP	-0.006 (-0.021, 0.010)	-0.014 (-0.041, 0.013)	0.591	0.000 (-0.017, 0.016)	-0.028 (-0.055, 0.000)	0.070
ORB	-0.004 (-0.019, 0.011)	-0.013 (-0.039, 0.014)	0.556	0.002 (-0.013, 0.018)	-0.031 (-0.058, -0.005)	0.020
TRI	-0.006 (-0.021, 0.009)	-0.004 (-0.031, 0.022)	0.907	-0.001 (-0.017, 0.015)	-0.022 (-0.048, 0.005)	0.161
PCG	-0.011 (-0.025, 0.004)	-0.022 (-0.048, 0.004)	0.434	-0.010 (-0.025, 0.006)	-0.038 (-0.064, -0.012)	0.049
rACC	-0.005 (-0.021, 0.010)	-0.024 (-0.051, 0.002)	0.205	-0.004 (-0.019, 0.012)	-0.033 (-0.060, -0.006)	0.048
RMFG	-0.008 (-0.022, 0.007)	-0.015 (-0.040, 0.009)	0.583	-0.003 (-0.018, 0.012)	-0.035 (-0.060, -0.010)	0.021
SFG	-0.008 (-0.022, 0.007)	-0.019 (-0.044, 0.006)	0.411	-0.002 (-0.017, 0.013)	-0.037 (-0.062, -0.012)	0.013
**Temporal lobe**						
Bankssts	-0.018 (-0.033, -0.002)	-0.005 (-0.033, 0.022)	0.425	-0.016 (-0.032, 0.000)	-0.021 (-0.048, 0.007)	0.774
EC	-0.002 (-0.018, 0.014)	-0.009 (-0.037, 0.019)	0.627	-0.004 (-0.021, 0.012)	-0.008 (-0.036, 0.020)	0.797
FG	-0.016 (-0.030, -0.001)	-0.032 (-0.057, -0.006)	0.261	-0.014 (-0.029, 0.001)	-0.040 (-0.066, -0.015)	0.064
ITG	-0.025 (-0.040, -0.011)	-0.015 (-0.040, 0.010)	0.457	-0.024 (-0.039, -0.009)	-0.028 (-0.053, -0.003)	0.794
MTG	-0.023 (-0.037, -0.009)	-0.015 (-0.040, 0.010)	0.587	-0.020 (-0.035, -0.006)	-0.032 (-0.057, -0.007)	0.388

PHG	-0.022 (-0.038, -0.005)	-0.008 (-0.036, 0.020)	0.391	-0.023 (-0.039, -0.006)	-0.011 (-0.040, 0.017)	0.477
STG	-0.028 (-0.042, -0.013)	-0.030 (-0.056, -0.005)	0.846	-0.025 (-0.041, -0.010)	-0.043 (-0.069, -0.017)	0.216
TTG	-0.018 (-0.034, -0.002)	-0.044 (-0.072, -0.016)	0.088	-0.017 (-0.033, 0.000)	-0.052 (-0.080, -0.024)	0.023
**Parietal lobe**						
IPL	-0.020 (-0.035, -0.005)	-0.004 (-0.031, 0.022)	0.277	-0.019 (-0.035, -0.004)	-0.016 (-0.042, 0.010)	0.830
isthmus cingulate	-0.007 (-0.022, 0.008)	-0.029 (-0.055, -0.003)	0.138	-0.006 (-0.022, 0.009)	-0.035 (-0.061, -0.009)	0.048
PoCG	-0.015 (-0.030, 0.001)	0.002 (-0.024, 0.029)	0.256	-0.013 (-0.029, 0.003)	-0.008 (-0.034, 0.019)	0.709
PCC	-0.006 (-0.022, 0.009)	-0.020 (-0.047, 0.006)	0.344	-0.006 (-0.022, 0.010)	-0.034 (-0.060, -0.007)	0.059
PCUN	-0.010 (-0.025, 0.005)	-0.028 (-0.054, -0.003)	0.199	-0.005 (-0.020, 0.010)	-0.039 (-0.065, -0.013)	0.017
SPL	-0.011 (-0.026, 0.004)	-0.021 (-0.047, 0.006)	0.526	-0.007 (-0.023, 0.009)	-0.032 (-0.059, -0.005)	0.089
SMG	-0.028 (-0.043, -0.013)	-0.027 (-0.053, -0.001)	0.962	-0.024 (-0.040, -0.009)	-0.039 (-0.065, -0.013)	0.309
**Occipital lobe**						
CUN	0.009 (-0.007, 0.024)	-0.013 (-0.040, 0.014)	0.154	0.010 (-0.006, 0.026)	-0.017 (-0.044, 0.010)	0.069
LOC	0.005 (-0.009, 0.020)	0.002 (-0.023, 0.027)	0.796	0.008 (-0.007, 0.023)	-0.001 (-0.026, 0.024)	0.519
LG	-0.003 (-0.018, 0.013)	-0.007 (-0.034, 0.021)	0.791	-0.002 (-0.019, 0.014)	-0.008 (-0.035, 0.020)	0.727
PCAL	0.002 (-0.014, 0.018)	-0.004 (-0.032, 0.024)	0.722	0.000 (-0.016, 0.017)	-0.004 (-0.032, 0.024)	0.793
**Insula lobe**						
INS	-0.032 (-0.047, -0.018)	-0.042 (-0.067, -0.017)	0.505	-0.032 (-0.047, -0.017)	-0.053 (-0.078, -0.028)	0.127

Abbreviations of the phenotype were shown in Table S1.

Table S7. Associations of TG and AIP with brain imaging phenotypes by WHR

Brain structures	TG		*P* for interaction	AIP		*P* for interaction
	Ideal	Poor		Ideal	Poor	
	β per IQR	*β* per IQR		*β* per IQR	*β* per IQR	
**Volume of subcortical structure**						
NAc	-0.008 (-0.026, 0.009)	-0.004 (-0.024, 0.015)	0.751	-0.014 (-0.032, 0.005)	0.003 (-0.017, 0.022)	0.221
AMYG	-0.005 (-0.024, 0.013)	0.018 (-0.003, 0.038)	0.093	-0.008 (-0.027, 0.012)	0.019 (-0.002, 0.040)	0.054
CN	-0.036 (-0.054, -0.017)	-0.037 (-0.058, -0.017)	0.907	-0.044 (-0.064, -0.025)	-0.03 (-0.051, -0.009)	0.316
HIP	-0.002 (-0.020, 0.016)	0.014 (-0.006, 0.034)	0.225	-0.009 (-0.028, 0.010)	0.018 (-0.003, 0.038)	0.046
GP	-0.031 (-0.049, -0.013)	-0.018 (-0.038, 0.002)	0.336	-0.038 (-0.058, -0.019)	-0.016 (-0.037, 0.005)	0.102
PUT	-0.022 (-0.038, -0.005)	-0.039 (-0.057, -0.021)	0.154	-0.028 (-0.045, -0.011)	-0.034 (-0.053, -0.016)	0.606
THA	-0.038 (-0.055, -0.022)	-0.019 (-0.037, 0.000)	0.105	-0.047 (-0.064, -0.029)	-0.015 (-0.034, 0.004)	0.011
**Volume of cortical structure**						
**Frontal lobe**						
cACC	-0.017 (-0.037, 0.002)	-0.026 (-0.047, -0.005)	0.539	-0.020 (-0.040, 0.000)	-0.030 (-0.052, -0.009)	0.479
cMFG	-0.010 (-0.028, 0.008)	-0.028 (-0.048, -0.007)	0.190	-0.015 (-0.034, 0.005)	-0.030 (-0.050, -0.009)	0.282
FP	0.037 (0.019, 0.056)	-0.003 (-0.023, 0.018)	0.003	0.037 (0.018, 0.056)	0.003 (-0.018, 0.024)	0.015
lOFC	-0.021 (-0.038, -0.004)	-0.025 (-0.044, -0.006)	0.741	-0.027 (-0.045, -0.009)	-0.023 (-0.043, -0.004)	0.757
mOFC	-0.026 (-0.043, -0.010)	-0.032 (-0.051, -0.014)	0.634	-0.031 (-0.048, -0.013)	-0.033 (-0.052, -0.014)	0.864
PCL	-0.006 (-0.024, 0.012)	-0.027 (-0.047, -0.007)	0.119	-0.011 (-0.031, 0.008)	-0.030 (-0.050, -0.009)	0.184
OP	-0.006 (-0.024, 0.012)	-0.013 (-0.033, 0.007)	0.609	-0.008 (-0.028, 0.011)	-0.013 (-0.033, 0.008)	0.766
ORB	0.003 (-0.015, 0.020)	-0.021 (-0.040, -0.001)	0.071	-0.004 (-0.022, 0.015)	-0.018 (-0.038, 0.002)	0.270
TRI	0.001 (-0.016, 0.019)	-0.018 (-0.038, 0.002)	0.131	-0.004 (-0.023, 0.014)	-0.017 (-0.037, 0.003)	0.352
PCG	-0.008 (-0.025, 0.009)	-0.023 (-0.042, -0.004)	0.239	-0.015 (-0.033, 0.003)	-0.026 (-0.046, -0.007)	0.378
rACC	-0.004 (-0.022, 0.014)	-0.019 (-0.039, 0.001)	0.276	-0.009 (-0.028, 0.010)	-0.017 (-0.037, 0.004)	0.562
RMFG	0.000 (-0.016, 0.017)	-0.026 (-0.044, -0.007)	0.034	-0.007 (-0.024, 0.011)	-0.024 (-0.042, -0.005)	0.176
SFG	-0.006 (-0.022, 0.011)	-0.020 (-0.039, -0.001)	0.245	-0.010 (-0.028, 0.008)	-0.020 (-0.039, -0.001)	0.426
**Temporal lobe**						
Bankssts	-0.012 (-0.030, 0.006)	-0.021 (-0.041, -0.001)	0.493	-0.016 (-0.035, 0.003)	-0.025 (-0.046, -0.005)	0.508
EC	-0.004 (-0.023, 0.015)	-0.003 (-0.024, 0.017)	0.978	-0.011 (-0.030, 0.009)	0.000 (-0.021, 0.021)	0.457
FG	-0.021 (-0.037, -0.004)	-0.021 (-0.040, -0.002)	0.963	-0.029 (-0.047, -0.011)	-0.018 (-0.037, 0.001)	0.381
ITG	-0.025 (-0.042, -0.009)	-0.022 (-0.041, -0.004)	0.805	-0.034 (-0.052, -0.017)	-0.020 (-0.039, -0.001)	0.261

MTG	-0.022 (-0.038, -0.005)	-0.024 (-0.042, -0.005)	0.858	-0.028 (-0.046, -0.011)	-0.026 (-0.045, -0.007)	0.833
PHG	-0.009 (-0.028, 0.011)	-0.034 (-0.055, -0.013)	0.071	-0.016 (-0.036, 0.004)	-0.031 (-0.053, -0.009)	0.303
STG	-0.025 (-0.042, -0.008)	-0.036 (-0.055, -0.017)	0.390	-0.032 (-0.050, -0.013)	-0.036 (-0.055, -0.016)	0.753
TTG	-0.023 (-0.041, -0.004)	-0.026 (-0.047, -0.005)	0.816	-0.031 (-0.051, -0.011)	-0.020 (-0.041, 0.002)	0.424
**Parietal lobe**						
IPL	-0.013 (-0.03, 0.005)	-0.026 (-0.045, -0.006)	0.304	-0.020 (-0.039, -0.002)	-0.025 (-0.045, -0.005)	0.726
isthmus cingulate	-0.011 (-0.029, 0.006)	-0.014 (-0.033, 0.006)	0.875	-0.017 (-0.036, 0.001)	-0.011 (-0.031, 0.009)	0.647
PoCG	-0.012 (-0.030, 0.006)	-0.012 (-0.032, 0.008)	0.999	-0.021 (-0.040, -0.002)	-0.009 (-0.029, 0.012)	0.376
PCC	-0.003 (-0.021, 0.015)	-0.019 (-0.039, 0.000)	0.219	-0.007 (-0.026, 0.012)	-0.024 (-0.044, -0.003)	0.218
PCUN	-0.006 (-0.023, 0.011)	-0.028 (-0.047, -0.009)	0.078	-0.013 (-0.031, 0.006)	-0.022 (-0.041, -0.002)	0.489
SPL	-0.003 (-0.021, 0.015)	-0.030 (-0.050, -0.010)	0.041	-0.009 (-0.028, 0.009)	-0.024 (-0.044, -0.003)	0.292
SMG	-0.024 (-0.041, -0.006)	-0.035 (-0.055, -0.016)	0.355	-0.029 (-0.048, -0.011)	-0.033 (-0.053, -0.013)	0.780
**Occipital lobe**						
CUN	0.004 (-0.014, 0.022)	0.002 (-0.019, 0.022)	0.833	0.001 (-0.018, 0.021)	0.003 (-0.017, 0.024)	0.901
LOC	0.006 (-0.011, 0.022)	0.000 (-0.018, 0.019)	0.672	0.001 (-0.016, 0.019)	0.004 (-0.015, 0.023)	0.814
LG	0.003 (-0.016, 0.021)	-0.013 (-0.033, 0.008)	0.256	-0.002 (-0.021, 0.018)	-0.011 (-0.032, 0.010)	0.504
PCAL	0.000 (-0.019, 0.019)	0.000 (-0.020, 0.021)	0.971	-0.003 (-0.023, 0.016)	0.002 (-0.019, 0.024)	0.682
**Insula lobe**						
INS	-0.033 (-0.050, -0.016)	-0.038 (-0.057, -0.019)	0.677	-0.040 (-0.057, -0.022)	-0.036 (-0.055, -0.017)	0.798

Abbreviations of the phenotype were shown in Table S1.

Table S8. Associations of TG and AIP with brain imaging phenotypes by healthy sleep pattern and regular physical activity

Brain structures	TG		*P* for interaction	AIP		*P* for interaction
	Ideal	Poor		Ideal	Poor	
	β per IQR	*β* per IQR		*β* per IQR	*β* per IQR	
**Healthy sleep pattern**						
**Volume of subcortical structure**						
NAc	-0.008 (-0.025, 0.008)	-0.004 (-0.023, 0.015)	0.717	-0.008 (-0.025, 0.009)	-0.003 (-0.023, 0.016)	0.691
AMYG	0.001 (-0.016, 0.019)	0.010 (-0.010, 0.031)	0.478	-0.001 (-0.019, 0.018)	0.012 (-0.009, 0.033)	0.322
CN	-0.041 (-0.058, -0.024)	-0.030 (-0.050, -0.010)	0.366	-0.041 (-0.059, -0.024)	-0.033 (-0.053, -0.012)	0.478
HIP	0.009 (-0.008, 0.026)	-0.001 (-0.021, 0.019)	0.410	0.004 (-0.014, 0.021)	0.002 (-0.018, 0.022)	0.903
GP	-0.019 (-0.037, -0.002)	-0.033 (-0.053, -0.013)	0.267	-0.023 (-0.041, -0.005)	-0.035 (-0.056, -0.015)	0.322
PUT	-0.028 (-0.043, -0.012)	-0.032 (-0.050, -0.013)	0.731	-0.030 (-0.046, -0.014)	-0.032 (-0.051, -0.014)	0.818
THA	-0.030 (-0.045, -0.014)	-0.029 (-0.047, -0.011)	0.918	-0.033 (-0.049, -0.017)	-0.031 (-0.049, -0.013)	0.842
**Volume of cortical structure**						
**Frontal lobe**						
cACC	-0.009 (-0.027, 0.009)	-0.038 (-0.059, -0.017)	0.027	-0.013 (-0.032, 0.005)	-0.041 (-0.062, -0.019)	0.036
cMFG	-0.014 (-0.031, 0.004)	-0.024 (-0.044, -0.004)	0.420	-0.016 (-0.034, 0.002)	-0.029 (-0.050, -0.009)	0.295
FP	0.021 (0.004, 0.039)	0.017 (-0.003, 0.038)	0.764	0.028 (0.010, 0.046)	0.013 (-0.008, 0.034)	0.241
lOFC	-0.026 (-0.042, -0.009)	-0.019 (-0.037, 0.000)	0.555	-0.029 (-0.045, -0.012)	-0.021 (-0.040, -0.002)	0.529
mOFC	-0.025 (-0.041, -0.009)	-0.035 (-0.053, -0.016)	0.388	-0.028 (-0.045, -0.012)	-0.036 (-0.055, -0.018)	0.476
PCL	-0.013 (-0.030, 0.004)	-0.018 (-0.038, 0.002)	0.696	-0.019 (-0.036, -0.001)	-0.021 (-0.042, -0.001)	0.820
OP	-0.001 (-0.018, 0.016)	-0.020 (-0.040, 0.000)	0.133	-0.004 (-0.021, 0.014)	-0.020 (-0.040, 0.000)	0.183
ORB	-0.009 (-0.025, 0.007)	-0.006 (-0.025, 0.013)	0.793	-0.012 (-0.029, 0.005)	-0.008 (-0.028, 0.011)	0.791
TRI	-0.001 (-0.018, 0.016)	-0.016 (-0.035, 0.004)	0.218	-0.004 (-0.021, 0.013)	-0.018 (-0.038, 0.001)	0.231
PCG	-0.019 (-0.035, -0.002)	-0.009 (-0.028, 0.010)	0.426	-0.022 (-0.039, -0.005)	-0.018 (-0.037, 0.002)	0.735
rACC	-0.006 (-0.023, 0.011)	-0.017 (-0.037, 0.003)	0.391	-0.008 (-0.026, 0.010)	-0.018 (-0.038, 0.002)	0.408
RMFG	-0.004 (-0.020, 0.011)	-0.021 (-0.039, -0.003)	0.141	-0.007 (-0.023, 0.009)	-0.025 (-0.044, -0.007)	0.102
SFG	-0.013 (-0.029, 0.003)	-0.011 (-0.029, 0.008)	0.852	-0.015 (-0.032, 0.001)	-0.014 (-0.032, 0.005)	0.877
**Temporal lobe**						
Bankssts	-0.023 (-0.040, -0.006)	-0.007 (-0.027, 0.013)	0.189	-0.028 (-0.046, -0.010)	-0.010 (-0.03, 0.010)	0.149
EC	-0.003 (-0.021, 0.015)	-0.005 (-0.025, 0.016)	0.888	-0.007 (-0.026, 0.011)	-0.004 (-0.025, 0.017)	0.782
FG	-0.025 (-0.041, -0.009)	-0.015 (-0.033, 0.004)	0.360	-0.031 (-0.047, -0.014)	-0.015 (-0.034, 0.004)	0.167
ITG	-0.022 (-0.038, -0.006)	-0.028 (-0.046, -0.009)	0.603	-0.027 (-0.044, -0.011)	-0.029 (-0.048, -0.01)	0.876

MTG	-0.023 (-0.039, -0.007)	-0.022 (-0.041, -0.004)	0.966	-0.028 (-0.044, -0.011)	-0.026 (-0.045, -0.008)	0.907
PHG	-0.016 (-0.034, 0.002)	-0.025 (-0.046, -0.004)	0.459	-0.022 (-0.041, -0.003)	-0.024 (-0.046, -0.003)	0.866
STG	-0.029 (-0.045, -0.012)	-0.032 (-0.051, -0.013)	0.769	-0.032 (-0.049, -0.015)	-0.035 (-0.055, -0.016)	0.765
TTG	-0.018 (-0.036, -0.001)	-0.032 (-0.053, -0.012)	0.265	-0.022 (-0.040, -0.003)	-0.032 (-0.053, -0.011)	0.434
**Parietal lobe**						
IPL	-0.020 (-0.037, -0.004)	-0.016 (-0.035, 0.003)	0.701	-0.024 (-0.041, -0.007)	-0.020 (-0.040, -0.001)	0.748
isthmus cingulate	-0.011 (-0.028, 0.005)	-0.014 (-0.033, 0.005)	0.803	-0.016 (-0.034, 0.001)	-0.012 (-0.031, 0.008)	0.687
PoCG	-0.013 (-0.030, 0.004)	-0.011 (-0.031, 0.009)	0.884	-0.016 (-0.033, 0.002)	-0.014 (-0.034, 0.006)	0.910
PCC	-0.002 (-0.019, 0.015)	-0.023 (-0.042, -0.003)	0.083	-0.006 (-0.024, 0.011)	-0.027 (-0.047, -0.007)	0.092
PCUN	-0.018 (-0.034, -0.001)	-0.013 (-0.032, 0.006)	0.680	-0.019 (-0.036, -0.003)	-0.013 (-0.032, 0.006)	0.584
SPL	-0.016 (-0.032, 0.001)	-0.013 (-0.033, 0.006)	0.866	-0.018 (-0.035, 0.000)	-0.014 (-0.034, 0.006)	0.751
SMG	-0.029 (-0.045, -0.012)	-0.029 (-0.048, -0.010)	0.985	-0.032 (-0.049, -0.015)	-0.029 (-0.048, -0.010)	0.785
**Occipital lobe**						
CUN	0.002 (-0.015, 0.019)	0.005 (-0.015, 0.025)	0.766	-0.001 (-0.019, 0.016)	0.007 (-0.013, 0.027)	0.499
LOC	0.005 (-0.011, 0.020)	0.001 (-0.017, 0.020)	0.776	0.004 (-0.013, 0.020)	0.001 (-0.017, 0.020)	0.841
LG	-0.004 (-0.021, 0.014)	-0.005 (-0.025, 0.015)	0.936	-0.009 (-0.027, 0.009)	-0.002 (-0.022, 0.019)	0.570
PCAL	0.002 (-0.016, 0.019)	-0.002 (-0.022, 0.019)	0.799	-0.002 (-0.020, 0.017)	0.000 (-0.021, 0.021)	0.886
**Insula lobe**						
INS	-0.038 (-0.054, -0.022)	-0.031 (-0.049, -0.013)	0.556	-0.041 (-0.057, -0.024)	-0.035 (-0.053, -0.016)	0.600
**Regular physical activity**						
**Volume of subcortical structures**						
NAc	-0.011 (-0.028, 0.006)	-0.001 (-0.019, 0.017)	0.381	-0.012 (-0.029, 0.006)	0.000 (-0.018, 0.019)	0.306
AMYG	0.010 (-0.008, 0.028)	-0.001 (-0.020, 0.019)	0.399	0.005 (-0.013, 0.024)	0.003 (-0.017, 0.023)	0.869
CN	-0.044 (-0.062, -0.026)	-0.028 (-0.047, -0.008)	0.186	-0.044 (-0.062, -0.025)	-0.031 (-0.051, -0.011)	0.308
HIP	0.002 (-0.015, 0.020)	0.008 (-0.011, 0.027)	0.640	0.001 (-0.017, 0.019)	0.005 (-0.014, 0.025)	0.714
GP	-0.022 (-0.040, -0.005)	-0.028 (-0.047, -0.009)	0.640	-0.027 (-0.045, -0.009)	-0.030 (-0.049, -0.010)	0.817
PUT	-0.032 (-0.048, -0.016)	-0.026 (-0.044, -0.009)	0.603	-0.034 (-0.050, -0.017)	-0.028 (-0.046, -0.010)	0.592
THA	-0.032 (-0.048, -0.016)	-0.026 (-0.043, -0.009)	0.581	-0.035 (-0.051, -0.018)	-0.029 (-0.047, -0.011)	0.608
**Volume of cortical structures**						
**Frontal lobe**						
cACC	-0.006 (-0.025, 0.013)	-0.040 (-0.060, -0.020)	0.009	-0.009 (-0.028, 0.010)	-0.043 (-0.064, -0.023)	0.008
cMFG	-0.013 (-0.030, 0.005)	-0.024 (-0.044, -0.005)	0.336	-0.015 (-0.034, 0.003)	-0.029 (-0.049, -0.010)	0.251
FP	0.022 (0.004, 0.040)	0.017 (-0.003, 0.036)	0.657	0.026 (0.007, 0.044)	0.016 (-0.004, 0.036)	0.442
lOFC	-0.020 (-0.037, -0.004)	-0.026 (-0.044, -0.008)	0.661	-0.021 (-0.038, -0.004)	-0.031 (-0.049, -0.013)	0.382

mOFC	-0.031 (-0.048, -0.015)	-0.026 (-0.044, -0.008)	0.651	-0.031 (-0.048, -0.014)	-0.032 (-0.050, -0.014)	0.945
PCL	-0.005 (-0.023, 0.013)	-0.028 (-0.047, -0.008)	0.066	-0.011 (-0.029, 0.007)	-0.031 (-0.050, -0.011)	0.107
OP	0.001 (-0.016, 0.019)	-0.021 (-0.041, -0.002)	0.062	0.001 (-0.017, 0.019)	-0.024 (-0.043, -0.004)	0.044
ORB	-0.006 (-0.023, 0.011)	-0.010 (-0.029, 0.008)	0.703	-0.009 (-0.026, 0.009)	-0.012 (-0.031, 0.007)	0.801
TRI	-0.002 (-0.019, 0.015)	-0.014 (-0.033, 0.005)	0.310	-0.005 (-0.023, 0.013)	-0.016 (-0.035, 0.003)	0.347
PCG	-0.010 (-0.027, 0.006)	-0.020 (-0.038, -0.002)	0.414	-0.014 (-0.031, 0.004)	-0.028 (-0.046, -0.009)	0.234
rACC	-0.002 (-0.019, 0.016)	-0.021 (-0.040, -0.002)	0.101	-0.003 (-0.021, 0.015)	-0.023 (-0.043, -0.004)	0.095
RMFG	-0.009 (-0.025, 0.007)	-0.014 (-0.031, 0.004)	0.689	-0.009 (-0.026, 0.007)	-0.020 (-0.038, -0.003)	0.325
SFG	-0.009 (-0.025, 0.007)	-0.015 (-0.033, 0.002)	0.581	-0.009 (-0.026, 0.007)	-0.021 (-0.039, -0.003)	0.297
**Temporal lobe**						
Bankssts	-0.013 (-0.031, 0.005)	-0.020 (-0.039, -0.001)	0.553	-0.018 (-0.036, 0.001)	-0.024 (-0.044, -0.004)	0.604
EC	0.007 (-0.012, 0.025)	-0.016 (-0.036, 0.004)	0.071	0.004 (-0.015, 0.023)	-0.017 (-0.038, 0.003)	0.087
FG	-0.018 (-0.034, -0.001)	-0.025 (-0.042, -0.007)	0.543	-0.019 (-0.036, -0.002)	-0.030 (-0.049, -0.012)	0.296
ITG	-0.024 (-0.041, -0.008)	-0.024 (-0.041, -0.006)	0.934	-0.026 (-0.043, -0.009)	-0.030 (-0.048, -0.012)	0.726
MTG	-0.023 (-0.039, -0.007)	-0.023 (-0.040, -0.005)	0.999	-0.027 (-0.044, -0.011)	-0.027 (-0.045, -0.009)	0.956
PHG	-0.018 (-0.036, 0.001)	-0.022 (-0.042, -0.002)	0.745	-0.020 (-0.039, -0.001)	-0.027 (-0.047, -0.006)	0.571
STG	-0.029 (-0.045, -0.012)	-0.032 (-0.050, -0.014)	0.777	-0.031 (-0.048, -0.014)	-0.036 (-0.055, -0.018)	0.658
TTG	-0.029 (-0.048, -0.011)	-0.018 (-0.038, 0.002)	0.353	-0.030 (-0.049, -0.012)	-0.020 (-0.041, 0.000)	0.431
**Parietal lobe**						
IPL	-0.026 (-0.043, -0.009)	-0.010 (-0.028, 0.009)	0.174	-0.030 (-0.047, -0.012)	-0.014 (-0.032, 0.005)	0.170
isthmus cingulate	-0.016 (-0.033, 0.001)	-0.008 (-0.026, 0.010)	0.498	-0.015 (-0.032, 0.003)	-0.014 (-0.033, 0.005)	0.970
PoCG	-0.009 (-0.026, 0.009)	-0.016 (-0.035, 0.003)	0.533	-0.009 (-0.027, 0.009)	-0.023 (-0.042, -0.003)	0.259
PCC	-0.005 (-0.022, 0.013)	-0.018 (-0.037, 0.001)	0.274	-0.007 (-0.025, 0.011)	-0.024 (-0.043, -0.005)	0.148
PCUN	-0.020 (-0.037, -0.003)	-0.010 (-0.028, 0.008)	0.396	-0.019 (-0.036, -0.002)	-0.014 (-0.033, 0.005)	0.662
SPL	-0.013 (-0.030, 0.004)	-0.017 (-0.036, 0.002)	0.749	-0.013 (-0.031, 0.005)	-0.019 (-0.039, 0.000)	0.629
SMG	-0.026 (-0.043, -0.009)	-0.032 (-0.050, -0.014)	0.622	-0.028 (-0.045, -0.011)	-0.035 (-0.053, -0.016)	0.566
**Occipital lobe**						
CUN	0.003 (-0.014, 0.021)	0.003 (-0.016, 0.022)	0.968	0.001 (-0.017, 0.019)	0.004 (-0.016, 0.023)	0.807
LOC	-0.004 (-0.021, 0.012)	0.012 (-0.005, 0.030)	0.134	-0.004 (-0.021, 0.012)	0.011 (-0.007, 0.029)	0.163
LG	-0.010 (-0.028, 0.008)	0.002 (-0.017, 0.022)	0.319	-0.010 (-0.029, 0.009)	-0.001 (-0.021, 0.019)	0.465
PCAL	0.002 (-0.016, 0.021)	-0.002 (-0.022, 0.017)	0.697	0.001 (-0.018, 0.020)	-0.003 (-0.024, 0.017)	0.721
**Insula lobe**						
INS	-0.035 (-0.051, -0.018)	-0.036 (-0.053, -0.018)	0.932	-0.038 (-0.055, -0.021)	-0.038 (-0.056, -0.020)	0.984

Abbreviations of the phenotype were shown in Table S1.

Table S9. Sensitivity analysis (1-4) of the main associations between TG and brain grey matter phenotypes

Brain structures	Sensitivity 1 [a]		Sensitivity 2 [b]		Sensitivity 3 [c]		Sensitivity 4 [d]	
	**β per IQR**	**P value FDR**	**β per IQR**	**P value FDR**	**β per IQR**	**P value FDR**	**β per IQR**	**P value FDR**
**Volume of subcortical structure**								
NAc	-0.008 (-0.022, 0.005)	0.289	-0.006 (-0.019, 0.007)	0.452	-0.001 (-0.014, 0.013)	0.938	-0.006 (-0.019, 0.008)	0.484
AMYG	0.006 (-0.009, 0.020)	0.533	0.005 (-0.009, 0.020)	0.526	0.006 (-0.009, 0.020)	0.522	0.005 (-0.009, 0.020)	0.535
CN	-0.039 (-0.054, -0.025)	<0.001	-0.036 (-0.050, -0.022)	<0.001	-0.035 (-0.049, -0.021)	<0.001	-0.036 (-0.050, -0.022)	<0.001
HIP	0.005 (-0.010, 0.019)	0.587	0.002 (-0.012, 0.016)	0.760	0.009 (-0.005, 0.023)	0.2902	0.005 (-0.008, 0.019)	0.519
GP	-0.027 (-0.042, -0.013)	0.001	-0.023 (-0.037, -0.009)	0.005	-0.022 (-0.036, -0.008)	0.008	-0.024 (-0.038, -0.010)	0.004
PUT	-0.032 (-0.045, -0.019)	<0.001	-0.030 (-0.043, -0.017)	<0.001	-0.025 (-0.038, -0.012)	0.001	-0.029 (-0.042, -0.016)	<0.001
THA	-0.027 (-0.040, -0.014)	<0.001	-0.030 (-0.042, -0.017)	<0.001	-0.027 (-0.040, -0.014)	<0.001	-0.028 (-0.041, -0.015)	<0.001
**Volume of cortical structure**								
**Frontal lobe**								
cACC	-0.023 (-0.038, -0.008)	0.009	-0.021 (-0.035, -0.006)	0.015	-0.019 (-0.034, -0.004)	0.030	-0.021 (-0.036, -0.006)	0.016
cMFG	-0.021 (-0.035, -0.007)	0.011	-0.020 (-0.034, -0.006)	0.014	-0.015 (-0.029, -0.001)	0.099	-0.017 (-0.032, -0.003)	0.035
FP	0.020 (0.005, 0.034)	0.015	0.020 (0.006, 0.034)	0.015	0.018 (0.004, 0.032)	0.035	0.018 (0.004, 0.032)	0.029
lOFC	-0.023 (-0.036, -0.010)	0.003	-0.025 (-0.038, -0.012)	0.001	-0.019 (-0.032, -0.006)	0.015	-0.021 (-0.035, -0.008)	0.005
mOFC	-0.028 (-0.041, -0.014)	<0.001	-0.029 (-0.042, -0.016)	<0.001	-0.026 (-0.039, -0.013)	0.001	-0.028 (-0.041, -0.015)	<0.001
PCL	-0.017 (-0.032, -0.003)	0.029	-0.015 (-0.029, -0.001)	0.062	-0.009 (-0.023, 0.005)	0.290	-0.015 (-0.029, -0.001)	0.068
OP	-0.010 (-0.024, 0.005)	0.247	-0.013 (-0.027, 0.001)	0.103	-0.007 (-0.021, 0.007)	0.436	-0.009 (-0.023, 0.005)	0.2813
ORB	-0.006 (-0.020, 0.007)	0.429	-0.012 (-0.025, 0.002)	0.115	-0.004 (-0.018, 0.009)	0.625	-0.007 (-0.020, 0.007)	0.428
TRI	-0.007 (-0.021, 0.007)	0.380	-0.012 (-0.026, 0.002)	0.115	-0.003 (-0.017, 0.010)	0.694	-0.008 (-0.021, 0.006)	0.365
PCG	-0.018 (-0.031, -0.004)	0.022	-0.017 (-0.031, -0.004)	0.023	-0.009 (-0.022, 0.005)	0.290	-0.014 (-0.027, 0.000)	0.078
rACC	-0.012 (-0.027, 0.002)	0.118	-0.013 (-0.027, 0.001)	0.087	-0.009 (-0.023, 0.005)	0.314	-0.010 (-0.024, 0.004)	0.217
RMFG	-0.011 (-0.024, 0.002)	0.150	-0.013 (-0.026, -0.001)	0.062	-0.009 (-0.022, 0.004)	0.283	-0.011 (-0.024, 0.002)	0.140
SFG	-0.012 (-0.025, 0.001)	0.110	-0.014 (-0.026, -0.001)	0.062	-0.010 (-0.023, 0.003)	0.258	-0.012 (-0.025, 0.001)	0.129
**Temporal lobe**								
Bankssts	-0.016 (-0.030, -0.001)	0.053	-0.017 (-0.031, -0.003)	0.035	-0.013 (-0.027, 0.002)	0.170	-0.015 (-0.029, -0.001)	0.068
EC	-0.004 (-0.018, 0.011)	0.680	-0.006 (-0.020, 0.008)	0.487	-0.002 (-0.016, 0.013)	0.884	-0.004 (-0.018, 0.011)	0.630
FG	-0.021 (-0.034, -0.008)	0.005	-0.023 (-0.036, -0.010)	0.002	-0.018 (-0.031, -0.005)	0.019	-0.021 (-0.034, -0.008)	0.005
ITG	-0.026 (-0.039, -0.013)	0.001	-0.025 (-0.038, -0.012)	0.001	-0.025 (-0.038, -0.012)	0.001	-0.023 (-0.036, -0.010)	0.002
MTG	-0.025 (-0.038, -0.012)	0.001	-0.022 (-0.035, -0.009)	0.002	-0.02 (-0.033, -0.007)	0.008	-0.022 (-0.035, -0.009)	0.004
PHG	-0.019 (-0.034, -0.004)	0.024	-0.021 (-0.036, -0.006)	0.014	-0.014 (-0.029, 0.000)	0.127	-0.019 (-0.034, -0.004)	0.029

STG	-0.029 (-0.042, -0.015)	<0.001	-0.031 (-0.044, -0.018)	<0.001	-0.025 (-0.038, -0.012)	0.001	-0.029 (-0.043, -0.016)	<0.001
TTG	-0.025 (-0.039, -0.010)	0.003	-0.028 (-0.042, -0.013)	0.001	-0.021 (-0.036, -0.007)	0.015	-0.024 (-0.039, -0.010)	0.004
**Parietal lobe**								
IPL	-0.020 (-0.034, -0.006)	0.011	-0.018 (-0.031, -0.004)	0.020	-0.017 (-0.030, -0.003)	0.039	-0.017 (-0.031, -0.004)	0.029
isthmus cingulate	-0.015 (-0.028, -0.001)	0.061	-0.014 (-0.027, 0.000)	0.066	-0.009 (-0.022, 0.005)	0.290	-0.012 (-0.025, 0.002)	0.140
PoCG	-0.014 (-0.028, 0.000)	0.068	-0.013 (-0.027, 0.001)	0.087	-0.010 (-0.024, 0.004)	0.283	-0.012 (-0.025, 0.002)	0.149
PCC	-0.013 (-0.027, 0.001)	0.110	-0.010 (-0.024, 0.003)	0.175	-0.006 (-0.020, 0.007)	0.473	-0.010 (-0.024, 0.004)	0.215
PCUN	-0.019 (-0.032, -0.005)	0.014	-0.017 (-0.030, -0.004)	0.023	-0.010 (-0.024, 0.003)	0.236	-0.015 (-0.029, -0.002)	0.050
SPL	-0.020 (-0.034, -0.006)	0.014	-0.015 (-0.028, -0.001)	0.062	-0.011 (-0.025, 0.003)	0.236	-0.015 (-0.029, -0.001)	0.068
SMG	-0.029 (-0.042, -0.015)	<0.001	-0.030 (-0.043, -0.016)	<0.001	-0.023 (-0.036, -0.010)	0.004	-0.028 (-0.042, -0.015)	<0.001
**Occipital lobe**								
CUN	0.003 (-0.011, 0.017)	0.724	0.003 (-0.011, 0.017)	0.738	0.004 (-0.010, 0.018)	0.625	0.003 (-0.011, 0.017)	0.668
LOC	0.001 (-0.012, 0.014)	0.852	0.002 (-0.011, 0.015)	0.760	0.005 (-0.008, 0.018)	0.522	0.004 (-0.009, 0.017)	0.630
LG	-0.005 (-0.019, 0.010)	0.587	-0.005 (-0.019, 0.010)	0.575	-0.003 (-0.017, 0.012)	0.783	-0.004 (-0.018, 0.010)	0.630
PCAL	0.003 (-0.011, 0.018)	0.680	0.000 (-0.015, 0.014)	0.987	0.001 (-0.014, 0.016)	0.914	0.001 (-0.014, 0.015)	0.918
**Insula lobe**								
INS	-0.035 (-0.048, -0.022)	<0.001	-0.037 (-0.050, -0.024)	<0.001	-0.032 (-0.044, -0.019)	<0.001	-0.034 (-0.047, -0.022)	<0.001

a analyses were restricted for white British Caucasians; **b** individuals with extremely small exposures (<1%) were excluded; **c** participants with extremely large exposures (>99%) were excluded; **d** analyses were performed with control for C-reactive protein.

Table S10. Sensitivity analysis (1-4) of the main associations between AIP and brain grey matter phenotypes

Brain structures	Sensitivity 1 [a]		Sensitivity 2 [b]		Sensitivity 3 [c]		Sensitivity 4 [d]	
	***β* per IQR**	**P value FDR**	***β* per IQR**	**P value FDR**	***β* per IQR**	**P value FDR**	***β* per IQR**	**P value FDR**
**Volume of subcortical structure**								
NAc	-0.008 (-0.022, 0.006)	0.346	-0.006 (-0.020, 0.008)	0.479	0.001 (-0.014, 0.015)	0.947	-0.005 (-0.020, 0.009)	0.530
AMYG	0.005 (-0.010, 0.020)	0.576	0.005 (-0.010, 0.020)	0.574	0.006 (-0.010, 0.021)	0.532	0.005 (-0.010, 0.020)	0.567
CN	-0.041 (-0.056, -0.026)	<0.001	-0.037 (-0.052, -0.022)	<0.001	-0.037 (-0.052, -0.022)	<0.001	-0.037 (-0.052, -0.023)	<0.001
HIP	0.003 (-0.012, 0.018)	0.731	0.001 (-0.014, 0.015)	0.912	0.009 (-0.006, 0.024)	0.307	0.004 (-0.011, 0.019)	0.649
GP	-0.030 (-0.045, -0.016)	<0.001	-0.026 (-0.040, -0.011)	0.002	-0.025 (-0.04, -0.010)	0.004	-0.027 (-0.041, -0.012)	0.002
PUT	-0.033 (-0.047, -0.020)	<0.001	-0.032 (-0.045, -0.018)	<0.001	-0.026 (-0.04, -0.013)	0.001	-0.031 (-0.044, -0.017)	<0.001
THA	-0.030 (-0.043, -0.016)	<0.001	-0.032 (-0.045, -0.019)	<0.001	-0.03 (-0.044, -0.016)	<0.001	-0.031 (-0.044, -0.017)	<0.001
**Volume of cortical structure**								
**Frontal lobe**								
cACC	-0.026 (-0.042, -0.010)	0.003	-0.024 (-0.040, -0.009)	0.005	-0.023 (-0.039, -0.007)	0.013	-0.025 (-0.040, -0.009)	0.006
cMFG	-0.024 (-0.039, -0.009)	0.004	-0.024 (-0.039, -0.009)	0.004	-0.018 (-0.033, -0.003)	0.046	-0.021 (-0.036, -0.006)	0.013
FP	0.022 (0.006, 0.037)	0.011	0.021 (0.006, 0.036)	0.010	0.020 (0.005, 0.036)	0.022	0.020 (0.005, 0.035)	0.019
lOFC	-0.026 (-0.040, -0.012)	0.001	-0.028 (-0.041, -0.014)	<0.001	-0.022 (-0.036, -0.008)	0.007	-0.024 (-0.038, -0.010)	0.002
mOFC	-0.030 (-0.044, -0.016)	<0.001	-0.032 (-0.045, -0.018)	<0.001	-0.029 (-0.043, -0.015)	<0.001	-0.031 (-0.045, -0.017)	<0.001
PCL	-0.022 (-0.037, -0.007)	0.008	-0.020 (-0.034, -0.005)	0.015	-0.013 (-0.028, 0.002)	0.1577	-0.019 (-0.034, -0.005)	0.019
OP	-0.011 (-0.026, 0.004)	0.209	-0.014 (-0.028, 0.001)	0.078	-0.008 (-0.023, 0.007)	0.3791	-0.010 (-0.025, 0.004)	0.224
ORB	-0.009 (-0.023, 0.006)	0.298	-0.014 (-0.028, 0.000)	0.067	-0.007 (-0.021, 0.008)	0.467	-0.009 (-0.023, 0.005)	0.277
TRI	-0.010 (-0.025, 0.004)	0.217	-0.015 (-0.029, 0.000)	0.061	-0.006 (-0.020, 0.009)	0.532	-0.010 (-0.025, 0.004)	0.214
PCG	-0.023 (-0.037, -0.008)	0.004	-0.023 (-0.036, -0.009)	0.004	-0.013 (-0.028, 0.001)	0.130	-0.019 (-0.033, -0.005)	0.019
rACC	-0.014 (-0.029, 0.001)	0.088	-0.015 (-0.029, 0.000)	0.060	-0.010 (-0.025, 0.005)	0.270	-0.012 (-0.026, 0.003)	0.163
RMFG	-0.014 (-0.027, 0.000)	0.071	-0.017 (-0.030, -0.003)	0.025	-0.012 (-0.026, 0.002)	0.156	-0.014 (-0.028, -0.001)	0.063
SFG	-0.015 (-0.028, -0.001)	0.056	-0.016 (-0.030, -0.003)	0.029	-0.012 (-0.026, 0.002)	0.155	-0.014 (-0.028, -0.001)	0.062
**Temporal lobe**								
Bankssts	-0.020 (-0.035, -0.005)	0.016	-0.021 (-0.036, -0.007)	0.009	-0.017 (-0.032, -0.002)	0.067	-0.019 (-0.034, -0.004)	0.020
EC	-0.005 (-0.020, 0.010)	0.576	-0.008 (-0.023, 0.007)	0.350	-0.003 (-0.019, 0.012)	0.734	-0.006 (-0.021, 0.009)	0.510
FG	-0.024 (-0.038, -0.010)	0.002	-0.026 (-0.04, -0.013)	0.001	-0.022 (-0.036, -0.008)	0.007	-0.025 (-0.038, -0.011)	0.002
ITG	-0.030 (-0.044, -0.016)	<0.001	-0.028 (-0.042, -0.015)	<0.001	-0.030 (-0.044, -0.016)	<0.001	-0.027 (-0.041, -0.013)	0.001
MTG	-0.030 (-0.043, -0.016)	<0.001	-0.027 (-0.04, -0.013)	<0.001	-0.025 (-0.039, -0.011)	0.002	-0.026 (-0.039, -0.012)	0.001
PHG	-0.022 (-0.038, -0.007)	0.011	-0.024 (-0.04, -0.009)	0.005	-0.017 (-0.033, -0.001)	0.070	-0.022 (-0.038, -0.007)	0.011

STG	-0.032 (-0.046, -0.018)	<0.001	-0.034 (-0.048, -0.021)	<0.001	-0.029 (-0.043, -0.014)	0.001	-0.033 (-0.047, -0.019)	<0.001
TTG	-0.026 (-0.042, -0.011)	0.003	-0.030 (-0.045, -0.015)	<0.001	-0.023 (-0.039, -0.008)	0.010	-0.026 (-0.041, -0.011)	0.002
**Parietal lobe**								
IPL	-0.024 (-0.038, -0.010)	0.003	-0.022 (-0.036, -0.008)	0.005	-0.021 (-0.035, -0.006)	0.013	-0.021 (-0.035, -0.007)	0.009
isthmus cingulate	-0.016 (-0.030, -0.002)	0.045	-0.016 (-0.030, -0.002)	0.038	-0.011 (-0.025, 0.004)	0.217	-0.014 (-0.028, 0.001)	0.088
PoCG	-0.017 (-0.032, -0.003)	0.036	-0.017 (-0.031, -0.002)	0.038	-0.013 (-0.028, 0.002)	0.156	-0.015 (-0.029, 0.000)	0.074
PCC	-0.017 (-0.031, -0.002)	0.041	-0.015 (-0.029, 0.000)	0.060	-0.010 (-0.025, 0.005)	0.265	-0.014 (-0.029, 0.000)	0.077
PCUN	-0.019 (-0.033, -0.005)	0.013	-0.018 (-0.032, -0.004)	0.018	-0.011 (-0.025, 0.003)	0.214	-0.016 (-0.030, -0.002)	0.039
SPL	-0.021 (-0.035, -0.006)	0.012	-0.016 (-0.030, -0.002)	0.044	-0.012 (-0.027, 0.003)	0.190	-0.016 (-0.031, -0.002)	0.051
SMG	-0.031 (-0.045, -0.017)	<0.001	-0.032 (-0.046, -0.018)	0.000	-0.025 (-0.039, -0.011)	0.003	-0.030 (-0.044, -0.016)	<0.001
**Occipital lobe**								
CUN	0.002 (-0.013, 0.017)	0.800	0.002 (-0.013, 0.016)	0.857	0.004 (-0.011, 0.019)	0.663	0.002 (-0.012, 0.017)	0.792
LOC	0.001 (-0.013, 0.015)	0.872	0.002 (-0.012, 0.015)	0.857	0.005 (-0.009, 0.019)	0.532	0.003 (-0.010, 0.017)	0.671
LG	-0.006 (-0.021, 0.009)	0.508	-0.006 (-0.021, 0.008)	0.463	-0.004 (-0.019, 0.011)	0.663	-0.006 (-0.021, 0.009)	0.536
PCAL	0.003 (-0.013, 0.018)	0.771	-0.001 (-0.017, 0.014)	0.870	0.000 (-0.015, 0.016)	0.990	0.000 (-0.015, 0.015)	0.974
**Insula lobe**								
INS	-0.038 (-0.052, -0.024)	<0.001	-0.040 (-0.053, -0.026)	<0.001	-0.035 (-0.049, -0.021)	<0.001	-0.038 (-0.051, -0.024)	<0.001

a analyses were restricted for white British Caucasians; **b** individuals with extremely small exposures (<1%) were excluded; **c** participants with extremely large exposures (>99%) were excluded; **d** analyses were performed with control for C-reactive protein.

Table S11. Sensitivity analysis (5-7) of the main associations between TG and brain grey matter phenotypes

Brain structures	Sensitivity 5 [a]		Sensitivity 6 [b]		Sensitivity 7 [c]	
	**β per IQR**	**P value FDR**	**β per IQR**	**P value FDR**	**β per IQR**	**P value FDR**
**Volume of subcortical structure**						
NAc	-0.007 (-0.020, 0.007)	0.406	-0.006 (-0.023, 0.012)	0.610	-0.006 (-0.020, 0.007)	0.433
AMYG	0.005 (-0.009, 0.019)	0.579	0.003 (-0.016, 0.022)	0.807	0.005 (-0.009, 0.019)	0.575
CN	-0.037 (-0.051, -0.022)	<0.001	-0.033 (-0.051, -0.014)	0.006	-0.036 (-0.050, -0.022)	<0.001
HIP	0.005 (-0.009, 0.018)	0.579	0.007 (-0.011, 0.026)	0.532	0.005 (-0.009, 0.019)	0.575
GP	-0.025 (-0.039, -0.011)	0.002	-0.023 (-0.041, -0.005)	0.037	-0.025 (-0.039, -0.011)	0.002
PUT	-0.029 (-0.042, -0.016)	<0.001	-0.030 (-0.047, -0.013)	0.006	-0.029 (-0.042, -0.016)	<0.001
THA	-0.030 (-0.042, -0.017)	<0.001	-0.025 (-0.041, -0.008)	0.017	-0.029 (-0.042, -0.017)	<0.001
**Volume of cortical structure**						
**Frontal lobe**						
cACC	-0.022 (-0.036, -0.007)	0.012	-0.030 (-0.050, -0.010)	0.017	-0.021 (-0.036, -0.007)	0.013
cMFG	-0.018 (-0.032, -0.004)	0.029	-0.025 (-0.044, -0.006)	0.029	-0.018 (-0.032, -0.004)	0.030
FP	0.020 (0.005, 0.034)	0.017	0.011 (-0.007, 0.030)	0.323	0.020 (0.005, 0.034)	0.018
lOFC	-0.023 (-0.036, -0.010)	0.002	-0.023 (-0.040, -0.006)	0.029	-0.023 (-0.036, -0.010)	0.003
mOFC	-0.029 (-0.042, -0.016)	<0.001	-0.026 (-0.043, -0.009)	0.017	-0.029 (-0.042, -0.016)	<0.001
PCL	-0.015 (-0.029, -0.001)	0.056	-0.020 (-0.039, -0.002)	0.058	-0.015 (-0.029, -0.001)	0.060
OP	-0.009 (-0.023, 0.005)	0.268	-0.012 (-0.030, 0.007)	0.305	-0.009 (-0.023, 0.005)	0.279
ORB	-0.008 (-0.021, 0.006)	0.329	-0.007 (-0.025, 0.010)	0.532	-0.008 (-0.021, 0.006)	0.331
TRI	-0.007 (-0.021, 0.007)	0.384	-0.013 (-0.032, 0.005)	0.223	-0.007 (-0.021, 0.006)	0.378
PCG	-0.015 (-0.028, -0.002)	0.054	-0.016 (-0.033, 0.002)	0.127	-0.015 (-0.028, -0.001)	0.058
rACC	-0.011 (-0.025, 0.003)	0.182	-0.019 (-0.038, -0.001)	0.068	-0.011 (-0.024, 0.003)	0.189
RMFG	-0.011 (-0.024, 0.001)	0.121	-0.019 (-0.036, -0.002)	0.058	-0.011 (-0.024, 0.001)	0.126
SFG	-0.012 (-0.025, 0.001)	0.114	-0.012 (-0.029, 0.006)	0.261	-0.012 (-0.025, 0.001)	0.113
**Temporal lobe**						
Bankssts	-0.017 (-0.031, -0.003)	0.041	-0.015 (-0.034, 0.003)	0.168	-0.016 (-0.030, -0.002)	0.047
EC	-0.004 (-0.018, 0.011)	0.657	-0.001 (-0.021, 0.019)	0.941	-0.004 (-0.018, 0.011)	0.656
FG	-0.021 (-0.034, -0.008)	0.005	-0.021 (-0.039, -0.004)	0.041	-0.021 (-0.034, -0.008)	0.006
ITG	-0.024 (-0.037, -0.011)	0.001	-0.025 (-0.042, -0.008)	0.020	-0.024 (-0.037, -0.011)	0.001
MTG	-0.023 (-0.036, -0.010)	0.002	-0.024 (-0.041, -0.007)	0.027	-0.023 (-0.036, -0.010)	0.002
PHG	-0.020 (-0.034, -0.005)	0.021	-0.024 (-0.043, -0.005)	0.037	-0.020 (-0.034, -0.005)	0.020
STG	-0.030 (-0.043, -0.017)	<0.001	-0.031 (-0.049, -0.014)	0.006	-0.030 (-0.043, -0.017)	<0.001

TTG	-0.024 (-0.039, -0.010)	0.004	-0.025 (-0.045, -0.006)	0.029	-0.024 (-0.039, -0.010)	0.004
**Parietal lobe**						
IPL	-0.019 (-0.032, -0.005)	0.017	-0.022 (-0.039, -0.004)	0.041	-0.018 (-0.032, -0.005)	0.018
isthmus cingulate	-0.012 (-0.026, 0.001)	0.114	-0.017 (-0.035, 0.001)	0.102	-0.012 (-0.026, 0.001)	0.113
PoCG	-0.012 (-0.026, 0.002)	0.122	-0.006 (-0.024, 0.012)	0.610	-0.012 (-0.026, 0.002)	0.126
PCC	-0.011 (-0.024, 0.003)	0.182	-0.020 (-0.038, -0.002)	0.058	-0.010 (-0.024, 0.003)	0.189
PCUN	-0.016 (-0.029, -0.002)	0.041	-0.020 (-0.037, -0.002)	0.058	-0.016 (-0.029, -0.002)	0.042
SPL	-0.015 (-0.029, -0.001)	0.064	-0.020 (-0.038, -0.002)	0.058	-0.015 (-0.028, -0.001)	0.064
SMG	-0.029 (-0.042, -0.016)	<0.001	-0.031 (-0.048, -0.013)	0.006	-0.029 (-0.042, -0.016)	<0.001
**Occipital lobe**						
CUN	0.003 (-0.011, 0.017)	0.669	0.005 (-0.013, 0.024)	0.644	0.003 (-0.011, 0.017)	0.664
LOC	0.003 (-0.010, 0.016)	0.657	0.001 (-0.017, 0.018)	0.941	0.003 (-0.010, 0.016)	0.656
LG	-0.005 (-0.019, 0.010)	0.579	-0.007 (-0.026, 0.011)	0.532	-0.004 (-0.018, 0.010)	0.620
PCAL	0.000 (-0.014, 0.015)	0.973	0.003 (-0.016, 0.022)	0.793	0.000 (-0.014, 0.015)	0.974
**Insula lobe**						
INS	-0.035 (-0.048, -0.022)	<0.001	-0.044 (-0.061, -0.027)	<0.001	-0.035 (-0.048, -0.022)	<0.001

a patients with pancreatitis were excluded; **b** analyses were performed after controlling for hypertension, heart attack and diabetes medications additionally; **c** analyses were conducted after controlling for antiplatelet or anticoagulant medications additionally.

Table S12. Sensitivity analysis (5-7) of the main associations between AIP and brain grey matter phenotypes

Brain structures	Sensitivity 5 [a]		Sensitivity 6 [b]		Sensitivity 7 [c]	
	β **per IQR**	**P value FDR**	***β*** **per IQR**	**P value FDR**	***β*** **per IQR**	**P value FDR**
**Volume of subcortical structure**						
NAc	-0.006 (-0.020, 0.008)	0.456	-0.006 (-0.024, 0.012)	0.601	-0.006 (-0.020, 0.008)	0.490
AMYG	0.005 (-0.010, 0.019)	0.615	0.002 (-0.018, 0.021)	0.887	0.005 (-0.010, 0.020)	0.610
CN	-0.038 (-0.053, -0.023)	<0.001	-0.032 (-0.052, -0.013)	0.007	-0.038 (-0.053, -0.023)	<0.001
HIP	0.003 (-0.012, 0.017)	0.729	0.005 (-0.014, 0.024)	0.727	0.003 (-0.011, 0.018)	0.721
GP	-0.028 (-0.043, -0.013)	0.001	-0.026 (-0.044, -0.007)	0.017	-0.028 (-0.043, -0.013)	0.001
PUT	-0.031 (-0.044, -0.018)	<0.001	-0.031 (-0.049, -0.014)	0.005	-0.031 (-0.044, -0.018)	<0.001
THA	-0.033 (-0.046, -0.019)	<0.001	-0.028 (-0.045, -0.011)	0.007	-0.032 (-0.045, -0.019)	<0.001
**Volume of cortical structure**						
**Frontal lobe**						
cACC	-0.025 (-0.041, -0.010)	0.004	-0.033 (-0.053, -0.012)	0.007	-0.025 (-0.040, -0.009)	0.005
cMFG	-0.022 (-0.036, -0.007)	0.010	-0.029 (-0.048, -0.009)	0.012	-0.021 (-0.036, -0.007)	0.010
FP	0.021 (0.007, 0.036)	0.010	0.013 (-0.006, 0.032)	0.266	0.021 (0.007, 0.036)	0.011
lOFC	-0.026 (-0.040, -0.012)	0.001	-0.025 (-0.043, -0.008)	0.017	-0.026 (-0.039, -0.012)	0.001
mOFC	-0.032 (-0.045, -0.018)	<0.001	-0.029 (-0.046, -0.011)	0.007	-0.032 (-0.045, -0.018)	<0.001
PCL	-0.020 (-0.035, -0.005)	0.014	-0.026 (-0.045, -0.007)	0.017	-0.020 (-0.034, -0.005)	0.016
OP	-0.010 (-0.025, 0.004)	0.206	-0.012 (-0.031, 0.007)	0.275	-0.010 (-0.025, 0.004)	0.213
ORB	-0.010 (-0.024, 0.004)	0.201	-0.009 (-0.027, 0.009)	0.403	-0.010 (-0.024, 0.004)	0.203
TRI	-0.010 (-0.024, 0.004)	0.214	-0.016 (-0.035, 0.002)	0.122	-0.010 (-0.024, 0.004)	0.213
PCG	-0.020 (-0.034, -0.006)	0.010	-0.021 (-0.039, -0.003)	0.038	-0.020 (-0.034, -0.006)	0.011
rACC	-0.012 (-0.027, 0.002)	0.131	-0.020 (-0.039, -0.001)	0.056	-0.012 (-0.027, 0.002)	0.134
RMFG	-0.015 (-0.028, -0.001)	0.054	-0.023 (-0.041, -0.006)	0.019	-0.014 (-0.028, -0.001)	0.055
SFG	-0.015 (-0.028, -0.001)	0.056	-0.016 (-0.034, 0.002)	0.113	-0.015 (-0.028, -0.001)	0.055
**Temporal lobe**						
Bankssts	-0.021 (-0.036, -0.006)	0.010	-0.021 (-0.040, -0.001)	0.056	-0.021 (-0.035, -0.006)	0.012
EC	-0.006 (-0.021, 0.009)	0.522	-0.004 (-0.024, 0.016)	0.758	-0.006 (-0.021, 0.009)	0.522
FG	-0.024 (-0.038, -0.011)	0.002	-0.024 (-0.042, -0.006)	0.019	-0.024 (-0.038, -0.010)	0.002
ITG	-0.028 (-0.042, -0.014)	<0.001	-0.028 (-0.046, -0.011)	0.007	-0.028 (-0.041, -0.014)	<0.001
MTG	-0.027 (-0.041, -0.014)	<0.001	-0.028 (-0.046, -0.011)	0.007	-0.027 (-0.041, -0.014)	<0.001
PHG	-0.023 (-0.038, -0.007)	0.009	-0.028 (-0.047, -0.008)	0.017	-0.023 (-0.038, -0.008)	0.009
STG	-0.034 (-0.047, -0.020)	<0.001	-0.036 (-0.054, -0.018)	0.002	-0.033 (-0.047, -0.020)	<0.001

TTG	-0.026 (-0.041, -0.011)	0.003	-0.028 (-0.047, -0.008)	0.017	-0.026 (-0.041, -0.011)	0.003
**Parietal lobe**						
IPL	-0.023 (-0.037, -0.008)	0.005	-0.026 (-0.044, -0.007)	0.017	-0.022 (-0.036, -0.008)	0.005
isthmus cingulate	-0.015 (-0.029, 0.000)	0.063	-0.020 (-0.038, -0.001)	0.056	-0.014 (-0.029, 0.000)	0.065
PoCG	-0.015 (-0.030, -0.001)	0.057	-0.009 (-0.027, 0.010)	0.447	-0.015 (-0.030, -0.001)	0.059
PCC	-0.015 (-0.029, -0.001)	0.062	-0.024 (-0.043, -0.006)	0.022	-0.015 (-0.029, 0.000)	0.065
PCUN	-0.017 (-0.031, -0.003)	0.032	-0.020 (-0.039, -0.002)	0.046	-0.017 (-0.031, -0.003)	0.032
SPL	-0.016 (-0.031, -0.002)	0.051	-0.021 (-0.040, -0.002)	0.046	-0.016 (-0.031, -0.002)	0.051
SMG	-0.031 (-0.045, -0.017)	<0.001	-0.032 (-0.051, -0.014)	0.005	-0.031 (-0.045, -0.017)	<0.001
**Occipital lobe**						
CUN	0.002 (-0.012, 0.017)	0.776	0.004 (-0.015, 0.023)	0.758	0.002 (-0.012, 0.017)	0.771
LOC	0.003 (-0.011, 0.016)	0.729	0.000 (-0.018, 0.018)	0.986	0.003 (-0.011, 0.016)	0.721
LG	-0.006 (-0.021, 0.009)	0.485	-0.008 (-0.028, 0.011)	0.489	-0.006 (-0.021, 0.009)	0.522
PCAL	-0.001 (-0.016, 0.014)	0.922	0.003 (-0.017, 0.022)	0.841	-0.001 (-0.016, 0.014)	0.922
**Insula lobe**						
INS	-0.038 (-0.052, -0.025)	<0.001	-0.047 (-0.064, -0.029)	<0.001	-0.038 (-0.052, -0.025)	<0.001

a patients with pancreatitis were excluded; **b** analyses were performed after controlling for hypertension, heart attack and diabetes medications additionally; **c** analyses were conducted after controlling for antiplatelet or anticoagulant medications additionally.
